# c-Fos Repression by Piwi Regulates *Drosophila* Ovarian Germline Formation and Tissue Morphogenesis

**DOI:** 10.1371/journal.pgen.1006281

**Published:** 2016-09-13

**Authors:** Jonathon D. Klein, Chunxu Qu, Xiaoyang Yang, Yiping Fan, Chunlao Tang, Jamy C. Peng

**Affiliations:** 1 Department of Developmental Neurobiology, St. Jude Children’s Research Hospital, Memphis, Tennessee, United States of America; 2 Department of Computational Biology, St. Jude Children’s Research Hospital, Memphis, Tennessee, United States of America; Harvard Medical School, UNITED STATES

## Abstract

*Drosophila melanogaster* Piwi functions within the germline stem cells (GSCs) and the somatic niche to regulate GSC self-renewal and differentiation. How Piwi influences GSCs is largely unknown. We uncovered a genetic interaction between Piwi and c-Fos in the somatic niche that influences GSCs. c-Fos is a proto-oncogene that influences many cell and developmental processes. In wild-type ovarian cells, c-Fos is post-transcriptionally repressed by Piwi, which destabilized the c-Fos mRNA by promoting the processing of its 3′ untranslated region (UTR) into Piwi-interacting RNAs (piRNAs). The c-Fos 3′ UTR was sufficient to trigger Piwi-dependent destabilization of a GFP reporter. Piwi represses c-Fos in the somatic niche to regulate GSC maintenance and differentiation and in the somatic follicle cells to affect somatic cell disorganization, tissue dysmorphogenesis, oocyte maturation arrest, and infertility.

## Author Summary

The *Drosophila melanogaster* ovary is consisted of germ cells differentiated from GSCs and ovarian somatic cells that provide structural support to the organ. Piwi is a ribonucleoprotein required for GSC maintenance and differentiation in the *Drosophila* ovary. Piwi does so by influencing GSC-autonomous mechanisms or the somatic niche signaling. The somatic niche is composed of cells adjacent to the GSC that signal to promote GSC functions. Piwi and piRNAs are also known to repress transposons. We found that Piwi genetically interacts with c-Fos in the somatic niche and represses c-Fos expression in the ovarian somatic cells. Piwi destabilizes the c-Fos mRNA by mediating the generation of piRNAs from its 3′ UTR. Piwi represses c-Fos in the somatic niche to promote GSC functions and in the somatic ovarian cells to influence cell organization, tissue morphogenesis, and egg production.

## Introduction

**Fig 1:**
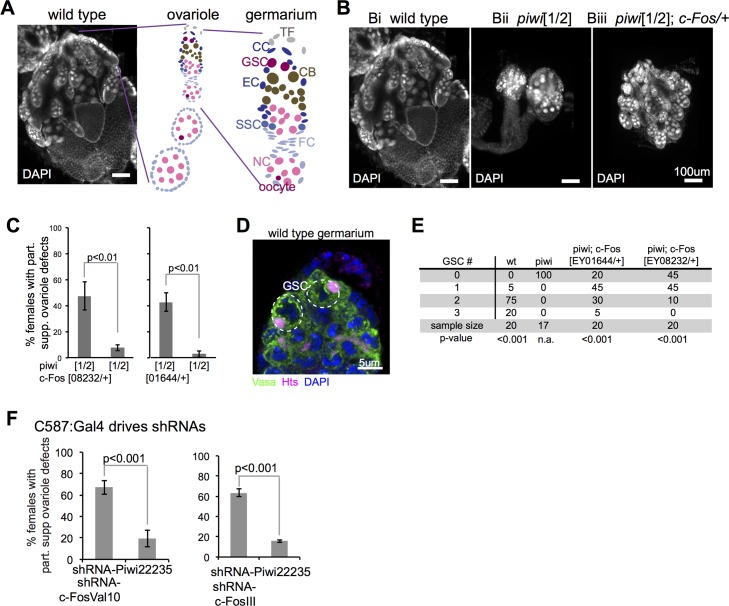
c-Fos reduction partially suppressed defects in *piwi* mutant ovaries. (A) DAPI-stained image of a wild-type ovary, a diagramed ovariole, and a diagramed germarium. Labeled cell types are, respectively, TF, terminal filament; CC, cap cell; GSC, germline stem cell; CB, cystoblast; EC, escort cell; SSC, somatic stem cell; FC, follicle cell; NC, nurse cell; oocyte. (B) Images of DAPI-stained ovaries from wild-type and mutant animals. (C) Quantification of Drosophila females with large (partially suppressed ovariole defects as shown in 1Biii) ovaries in piwi[1/2] and c-Fos/+;piwi[1/2]. (D) Vasa (green) and Hts (magenta) IF and DAPI (blue) staining of a wild-type germarium, with white dashed circles around GSCs. (E) Quantification of GSCs per germarium. (F) Quantification of Drosophila females with large (partially suppressed ovariole defects as shown in 1Biii) ovaries in animals with C587:Gal4 (escort cell-specific) driving Piwi and c-Fos shRNAs. Error bars represent standard deviation, and the chi-square test was used for statistical comparison.

Two major stem cell types are present in the *Drosophila* ovary: germline stem cells (GSCs) and somatic stem cells. Somatic stem cells differentiate into somatic follicle cells that provide structural support of the egg chamber. GSCs differentiate into germ cells, which become nurse cells or oocytes (Fig 1A). The somatic niche (or the GSC microenvironment) promotes GSC maintenance via signaling factors such as dpp/BMP [1, 2]. Piwi in GSCs and in the somatic niche promotes GSC maintenance and differentiation [3–7]. *piwi* mutant flies have no or markedly underdeveloped ovaries. Phenotypic studies of *piwi* mutant mosaic clones suggest that Piwi also affects oogenesis [3, 4, 8]. A genome-wide screen identified genetic interactors of Piwi [9], and follow-up studies revealed that Piwi interacts with Corto and Polycomb Group proteins to regulate GSCs [10, 11]. However, the molecular mechanism by which Piwi regulates GSC maintenance and differentiation is not well understood.

Piwi associates with Piwi-interacting RNAs (piRNAs), which are small (26–32 nt) RNAs that preferentially contain uridine as the first residue [12] and possess a 2-O-methylation site at the 3′ end [13]. The biogenesis of piRNAs and their repression of transposon activities to safeguard germline genome integrity have been extensively studied [14–16]. Primary piRNAs are generated from long, single-stranded precursor RNAs and undergo amplification through the ping-pong pathway to generate secondary piRNAs [17, 18]. Although the molecular factors and mechanisms that control the amplification of secondary piRNAs are well characterized, primary piRNA biogenesis mechanism is less understood.

We followed-up on a previous genetic screening analysis [9], and found that the proto-oncogene *c-Fos* is involved in Piwi-mediated regulation of GSCs. As part of the activator protein-1 complex, c-Fos regulates genes that control cell proliferation, differentiation, and survival [19–21]. We found that Piwi-mediated repression of *c-Fos* in the somatic niche regulates GSC maintenance and differentiation. Further, we reveal that the c-Fos mRNA serves as a piRNA precursor that is negatively regulated by Piwi, and this destabilization promotes somatic cell organization during ovarian tissue morphogenesis and animal fertility.

## Results

### *c-Fos* reduction partially rescues germline development and GSCs in *piwi* mutant

A genetic screen previously identified genomic regions whose heterozygous deficiency partially suppressed ovariole developmental defects in *piwi* mutant flies [9]. To follow up on this screen, we analyzed 31 fly lines with well-characterized genetic mutations located within these genomic regions. Each mutation was analyzed in flies with trans-heterozygous *piwi* mutant alleles 1 and 2 [3, 22] at day 4 post eclosion to ensure approximate developmental equivalency. Further, wild-type and *piwi* mutant ovaries had similar germ cell to somatic cell ratios, as revealed by a comparison of Vasa (germ cells) and Tj (somatic cells) mRNA and protein levels (S1A and S1B Fig).

Small (approximately less than 200 μm) ovaries with few ovarioles, similar to *piwi* mutant in Fig 1Bii, were categorized as having ovary defects. Large (approximately larger than 200 μm) ovaries with greater than 10 ovarioles, similar to *piwi*; *c-Fos*/+ mutant in Fig 1Biii, were categorized as having partially suppressed ovariole defects. We identified 2 independent P-element insertions in *c-Fos*, EY01644 and EY08232 alleles, that partially suppressed the ovariole defects in *piwi*[1/2] mutants, homozygous *piwi*[1/1], and heterozygous *piwi*[2/06839] mutants (Figs 1B and 1C and S1C). It was previously shown that transgenic expression of c-Fos cDNA rescued the homozygous lethal phenotype of the EY01644 mutant allele [23]. We found that the homozygous lethality of the EY08232 allele is also rescued by transgenic expression of c-Fos cDNA (S1D Fig). Thus, genetic reduction of c-Fos partially suppresses the ovariole defects in *piwi* mutant flies.

Each *Drosophila* ovary is composed of 18–22 ovarioles, which are spatially organized to house germline development and maturation [24]. The germarium at the tip of each ovariole contains GSCs and somatic stem cells [25]. In wild-type flies, GSCs are defined by their apical position at the germarium, the cytoplasmic expression of Vasa, the localization of Hts to the spectrosome, (a GSC-specific form of the fusome [24, 26]; Fig 1C), and the ability to differentiate into germ cells in egg chambers. We used this functional definition to quantify the number of GSCs in wild type, *piwi* single and *piwi;c-Fos*/+ double mutant fly lines. We found that wild type flies contained 1–3 GSCs/germarium. Both of the *piwi; c-Fos/+* double mutant lines displayed higher numbers of GSCs/germarium than the *piwi* mutant (Fig 1D and 1E). These findings indicate that *c-Fos* mutations partially suppress GSC loss in *piwi* mutant ovaries.

Next, we investigated the cell type(s) that underlie the suppressive effect of *c-Fos* mutations in the ovaries of *piwi* mutant flies. We used the Gal4/UAS system to drive cell type-specific expression of small hairpin RNAs (shRNAs) targeting *piwi* (22235, 33724) and/or *c-Fos* (II, Val10, and III; S1E and S1F Fig) [27–29]. Nos:Gal4, Tj:Gal4 and C587:Gal4 drivers were used to induce shRNA expression in ovarian germ, somatic, and escort cells, respectively.

We found that flies with reduced *piwi* expression in germ cells lacked ovarioles, as expected; however, this phenotype was not suppressed by loss of *c-Fos* (S1G Fig). In contrast, flies with reduced *piwi* expression in ovarian somatic or escort cells displayed ovariole defects and reduction of c-Fos in the respective cells did partially suppress these ovariole defects (Fig 1F and S1H Fig). These results indicate that Piwi interacts with c-Fos in the somatic niche to regulate germline development.

To investigate whether c-Fos function in GSCs or germ cells affects fertility, we used Nos:Gal4 driving *c-Fos* shRNAs to deplete c-Fos specifically in the germ cells. We found that these flies laid significantly fewer eggs than control animals (S2A Fig). This finding suggests that *c-Fos* in the GSCs and germ cells is required for normal fertility. We examined various cellular processes that are important for germ cells, such as meiotic double-stranded DNA break repair, ring canal structure, oocyte axis patterning, and detected no significant difference between flies depleted of *c-Fos* and wild-type controls (S2B–S2I Fig). These results suggest that *c-Fos* is required for normal speed of germ cell maturation and egg production. Thus, c-Fos in the GSCs and germ cells is required for female fecundity.

Germaria of the *piwi* mutants contained more spectrosomes than those of the wild type (S3A Fig), consistent with previous findings [5, 6]. Unexpectedly, the average numbers of germ cells containing spectrosomes per germarium were significantly higher in the *piwi; c-Fos/+* double mutants than in the *piwi* mutant (S3A and S3B Fig). This is likely caused by differentiation defects, which would result in little to no egg chamber formation. To determine whether this was the case, we quantified the number of egg chambers per ovariole. We found that the percentage of ovarioles with 3 or more egg chambers and the average number of egg chambers per ovariole were significantly higher in the *piwi;c-Fos*/+ double mutants than the *piwi* mutant (S3C and S3D Fig). These findings indicate that germ cell differentiation is partially rescued by the *c-Fos*/+ mutations in the *piwi* mutant ovaries. The combination of increased undifferentiated germ cells with partially rescued egg chamber formation in the *piwi;c-Fos*/+ double mutants likely reflect that *piwi;c-Fos*/+ double mutants have two populations of germ cells: a population that continually proliferates but cannot differentiate, and a different population that continually proliferate and differentiate. Altogether these results suggest that c-Fos functionally interacts with Piwi to affect the maintenance and differentiation of GSCs.

c-Fos is known to affect dpp signaling during embryogenesis [30] and in follicle cells of late-stage egg chambers [31]. Therefore, we examined whether c-Fos affects GSCs via dpp signaling. dpp/BMP signaling in the somatic niche induces Mad phosphorylation, which in turn represses the differentiation factor bag-of marbles (bam) to promote GSC maintenance [2, 32, 33]. We used immunofluorescence to quantify the number of phosphorylated Mad (pMad)-positive germ cells (S4A Fig). The identification of GSCs in *piwi* mutants is complicated by the presence of some germ cells containing spectrosomes but not pMad (S4A Fig). However, we found no significant difference in the number of pMad-positive germ cells between *piwi* and *piwi;c-Fos*/+ double mutant ovarian tissues (S4B Fig). These findings were confirmed in flies with somatic cell-specific RNAi knockdown of *piwi* and/or *c-Fos* (S4C Fig). Further, we found that mutations of Jun kinase, which phosphorylates c-Fos as part of JUNK signaling, did not suppress the ovariole defects in *piwi* mutants (S4D Fig). These findings suggest that c-Fos does not affect Piwi-mediated regulation of GSC function via dpp/BMP or JNK signaling pathways.

### Piwi-mediated repression of *c-Fos*

**Fig 2:**
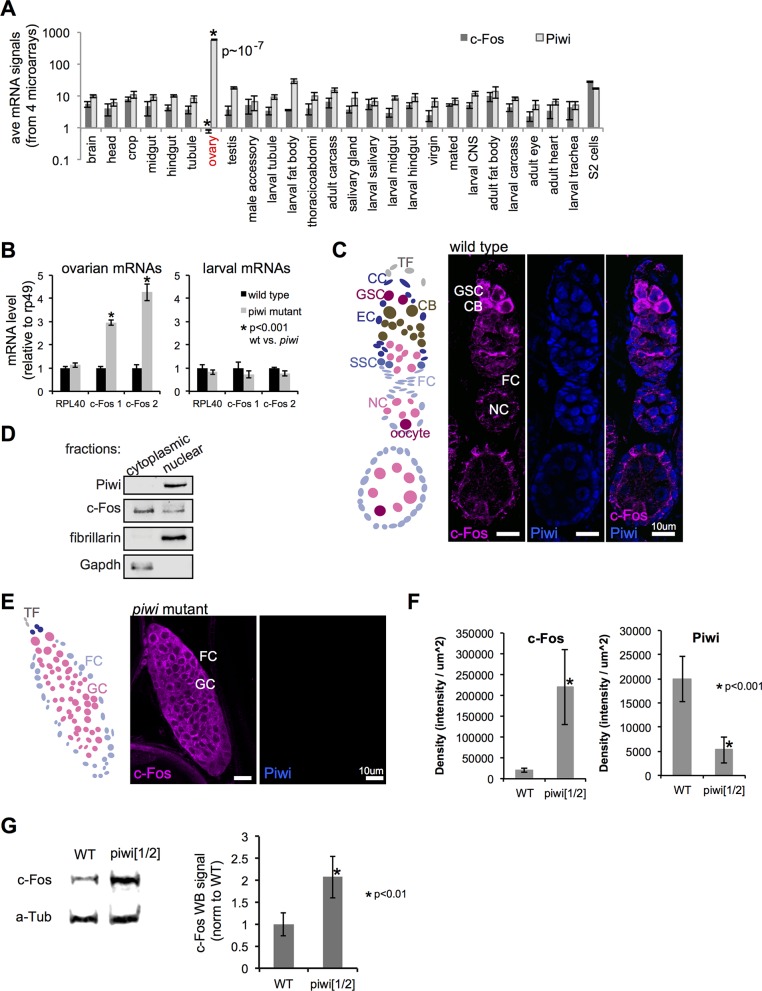
Piwi represses c-Fos in ovarian somatic and germ cells. (A) Average mRNA levels of *c-Fos* and *Piwi* across 26 distinct adult and larval tissue types, from 4 replicate data with Affymetrix Dros Genome 2 chips [34]. The Grubb’s test shows that in the ovary c-Fos and Piwi are strongly negatively correlated in the ovary (*P* < 10^−7^). (B) RT-qPCR quantitation of RPL40 and c-Fos (standardized by rp49) mRNAs in ovarian cells or larval cells from the wild-type and *piwi*[1/2] mutant. c-Fos 1 and c-Fos 2 are 2 different sets of RT-qPCR primers used to quantitate the c-Fos cDNA. c-Fos (magenta) and Piwi (blue) IF of (C) a wild-type or (E) *piwi*[1/2] ovariole. Labeled cell types are, respectively, TF, terminal filament; CC, cap cell; GSC, germline stem cell; CB, cystoblast; EC, escort cell; SSC, somatic stem cell; FC, follicle cell; NC, nurse cell; oocyte. (D) Piwi, c-Fos, fibrillarin, and Gapdh WB analysis of cytoplasmic and nuclear fractionation of wild-type ovarian cells. (F) Quantitation of c-Fos and Piwi IF signals (intensity/μm^2^) in wild-type and *piwi*[1/2] mutant ovarian cells. (G) Left: representative c-Fos and α-tubulin WB of wild-type and *piwi* ovarian extract. Right: quantitation of data from triplicate c-Fos WB using α-tubulin for normalization. Error bars represent standard deviation, and the Student’s *t* test was used for statistical comparison.

The finding that *c-Fos* reduction can partially suppress *piwi* mutant phenotypes suggests that Piwi represses *c-Fos* in ovaries. We collected c-Fos and Piwi expression data across 26 *Drosophila* tissue and cell types from FlyAtlas [34]. We noticed that the expression of c-Fos and Piwi were anti-correlative specifically in the ovary, where c-Fos was expressed at a significantly low level and Piwi at a significantly high level (Fig 2A; *p* = 1.485 × 10^−7^ by the Grubbs test). We analyzed c-Fos expression by RT-qPCR using 2 different primer sets and found that the c-Fos mRNA level in *piwi*[1/2] mutant ovarian cells was significantly higher than that in wild-type ovaries (Fig 2B). In contrast, c-Fos mRNA levels did not differ between *piwi* mutant and wild-type larval cells (Fig 2B). To determine if a developmental stage difference between wild-type and *piwi* mutant ovaries might underlie altered *c-Fos* expression, we analyzed *ovo* mutant ovaries, which have similar defects to *piwi* mutant ovaries [35, 36]. Although the *ovo* mutant ovaries displayed increased RPL40 and c-Fos mRNA levels (S5A Fig), the increase in c-Fos mRNA observed in *piwi* mutant ovaries was higher and more specific (RPL40 did not increase in *piwi* mutant). To determine if Piwi affects transcription of the *c-Fos* locus, we examined 3 replicate data sets of RNA polymerase II ChIP-seq in wild-type and *piwi* mutant ovarian cells [11]. We found that the *piwi* mutations did not affect RNA polymerase II binding to the *c-Fos* promoter, which was confirmed by ChIP-qPCR (S5B Fig). These data suggest that Piwi does not affect transcription of *c-Fos*. Thus, data from FlyAtlas, RT-qPCR, and chromatin immunoprecipitation (IP)-qPCR suggest that *c-Fos* repression is Piwi-dependent, post-transcriptional, and specific to the ovary.

We also evaluated c-Fos protein levels by immunofluorescence (IF) and Western blotting (WB). The specificity of the antibodies used for IF and WB was confirmed by RNAi-mediated *c-Fos* depletion (S1E and S1F Fig). c-Fos protein levels were higher in GSCs and the adjacent cystoblasts than in somatic and other germ cells, as shown by IF and confocal microscopy (using the same imaging parameters for the wild type and the *piwi* mutant; Fig 2C). Piwi was present in all nuclei of the ovary, and c-Fos localized to both the cytoplasm and the nucleus (Fig 2C and 2D). Further, c-Fos protein levels were high in all cells in the *piwi* mutant ovarian cells (Fig 2E). Quantitation of the IF signals indicated that c-Fos was significantly increased, whereas Piwi was significantly decreased, in *piwi* mutant cells (Fig 2F). Indeed, we observed a 2-fold increase in c-Fos protein levels in *piwi* mutant compared to the wild type ovaries (Fig 2*G*). Together, these data suggest that Piwi represses *c-Fos* expression in in the ovarian somatic cells.

### The c-Fos 3ʹ untranslated region (UTR) is a piRNA precursor that generates mature, primary piRNAs

**Fig 3:**
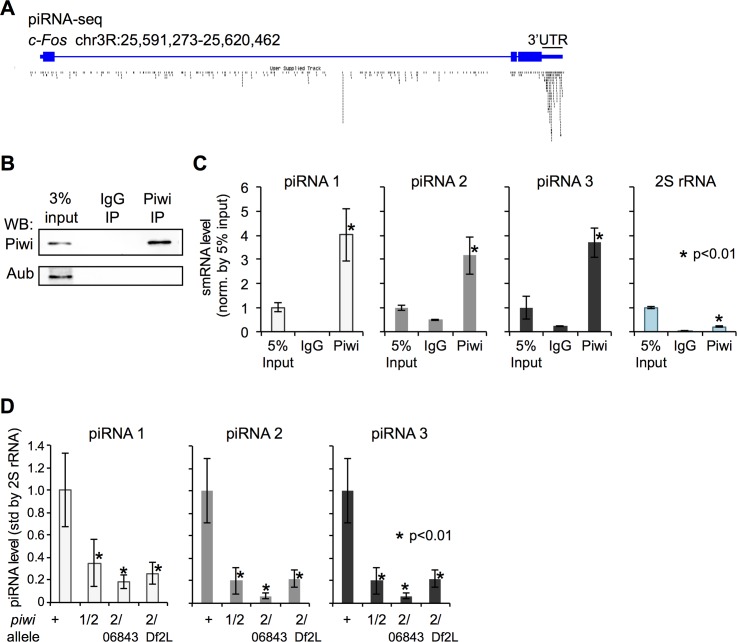
The 3′ UTR from the c-Fos mRNA is a primary piRNA precursor. (A) Diagram of the *c-Fos* locus. Published piRNA sequences are represented by black dashes. (B) Piwi and Aub WB of Piwi and IgG IP from wild-type ovarian cells. (C) TaqMan RT-qPCR quantitation of putative piRNAs 1–3 (unique to the c-Fos 3′ UTR) and 2S rRNA. The 5% input serves as normalization. (D) TaqMan RT-qPCR analysis of putative piRNAs 1–3 in ovarian cells from wild-type and 3 *piwi* mutant allele combinations. 2S rRNA served as normalization. Values represent means of 3 RT reactions, with error bars representing standard deviation and the Student’s *t* test used for statistical comparison.

We next examined whether piRNAs are involved in Piwi-mediated repression of *c-Fos*. We aligned published piRNA sequences p and found that 429 piRNA sequences uniquely mapped to the entire *c-Fos* locus. Of these 429 piRNA sequences, 135 mapped to the 3′ UTR (Fig 3A). A binomial test to determine the significance of unique piRNA enrichment at the c-Fos 3′ UTR yielded a *p*-value of 2.2 × 10^−16^, indicating significantly higher enrichment of piRNAs at the 3′ UTR than the rest of the locus. Sequences of the entire c-Fos 3′ UTR or the piRNAs do not exhibit homology to retrotransposon sequences. The published studies [12, 37–39] and our study have all used size selection of approximately 26-30nt for sequencing small non-coding RNAs. This size selection excludes the presence of RNAs outside this size range; thus we cannot rule out a potential scenario of other non-coding RNAs originated from or targeting the c-Fos 3′ UTR. Nevertheless, we found that 126/135 piRNAs were in the sense orientation, consistent with primary piRNAs [12, 17, 40]. These data suggest that the c-Fos 3′ UTR is a primary piRNA precursor.

Next, we validated the putative piRNAs unique to the c-Fos 3′ UTR. Because of the relative low abundance of these piRNAs (S6A Fig), we used a stem-loop RT primer to amplify these piRNAs for RT-qPCR [41]. We designed TaqMan probes that are specific to these RT-PCR products and do not recognize the longer piRNA precursors (S6B Fig). TaqMan assays were designed to detect 3 predicted piRNAs unique to the c-Fos 3′ UTR, termed piRNA1-3 (S6C Fig). We detected expression of these piRNAs in fly ovaries, which was reduced upon depletion of c-Fos by RNAi (S6D Fig), confirming specificity of the TaqMan assays. These data suggest that piRNAs are specifically generated from the c-Fos 3’UTR in fly ovaries.

To determine if c-Fos piRNAs associate with Piwi, we performed immunoprecipitation (IP) experiments. We IP’d Piwi and IgG from *Drosophila* ovarian cell extracts and detected Piwi but not Aub (the closest homolog of Piwi) in Piwi IPs, confirming the specificity of the Piwi IP (Fig 3B). We radioactively end-labeled RNAs that co-precipitated with Piwi and IgG, and observed an enrichment of small RNAs in the Piwi IP (S6E Fig). We performed TaqMan RT-qPCR and found that c-Fos piRNAs 1–3 were enriched in the Piwi IP (Fig 3C). In comparison, 2S rRNA enrichment in the Piwi IP is significantly lower (Fig 3C). We also analyzed small RNAs purified from the ovaries of *piwi* mutants and wild-type flies and found significantly lower levels of piRNAs 1–3 in the three *piwi* mutant lines than in the wild type (Fig 3D). The association of c-Fos piRNAs with Piwi and the requirement of Piwi for their biogenesis/stability provide additional biological support for these computationally identified piRNAs.

Previous studies utilized an *in vitro* cell line, ovarian somatic cells (OSC), to examine the effect of Piwi and piRNA biogenesis factors on OSC transcriptomes [42, 43]. c-Fos FPKM levels from Sienski et al. and Ohtani et al. in OSCs are (i) significantly higher than that in wild type ovarian cells, (ii) unaffected by depletion of biogenesis factors Piwi, Armi, or Mael by siRNAs, and (iii) of similar c-Fos level in the *piwi* mutant ovarian cells from our data (S7A Fig). Yet, piRNAs from c-Fos are detected in the OSCs. One explanation is that OSCs and the ovarian cells differ in genes involved in germline development: down-regulated genes in the ovarian cells are enriched in cell adhesion, motion, and morphogenesis, while upregulated genes are enriched in reproductive processes, game production, eggshell formation, oogenesis, and cytoplasm organization (S7B and S7C Fig). Our findings by FlyAtlas gene expression profiling, RT-qPCR, IF, and WB support the conclusion that Piwi represses c-Fos in the ovarian cells. The molecular differences observed between *Drosophila* OSCs and ovarian cells (S7B and S7C Fig) suggest that Piwi requires a yet-identified mechanism/factor(s) present in the ovarian cells but absent in OSCs to mediate developmentally important gene regulation.

### The 3′ UTR of c-Fos is sufficient to induce gene repression

**Fig 4:**
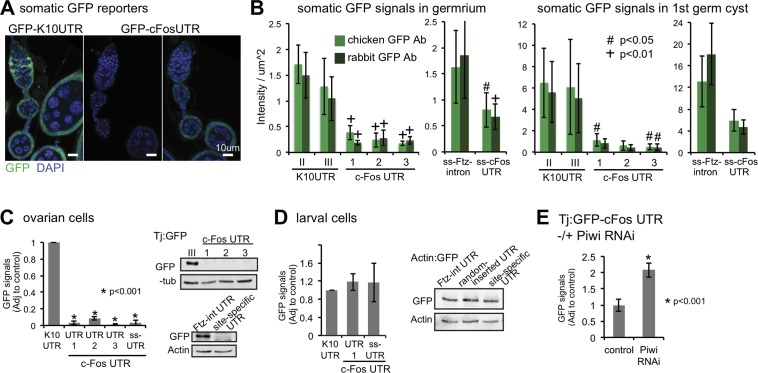
The 3′ UTR of c-Fos mRNA induces gene repression in the *Drosophila* ovarian cells. (A) Confocal images of GFP (green) IF of ovaries from Tj:Gal4; GFP-UTR (K10 control or c-Fos). (B) Quantitation of GFP IF signals in germarium or first germ cyst of Tj:Gal4; GFP-UTR. Quantitation of triplicate GFP WB and representative WB from (C) Tj:Gal4; GFP-UTR, (D) Actin:Gal4; GFP-UTR larval cells, or (E) Tj:Gal4; GFP-c-Fos UTR with or without Piwi shRNA.GFP-K10UTR was the control for (C) and (D). GFP-K10UTRs II-III and GFP-c-Fos UTRs 1–3 were generated by random integration. GFP-ss-Ftz-intron and GFP-ss-c-FosUTR were generated by PhiC31-mediated, site-specific integration. Error bars represent standard deviation, and the Student’s *t* test was used for statistical comparison.

To determine if the c-Fos 3’UTR is sufficient to repress gene expression in fly ovaries, we generated transgenic *Drosophila* expressing GFP reporters of the c-Fos 3ʹ UTR either by random site integration (GFP-c-Fos-UTR-1,-2, -3 on the UASp vector) or PhiC31-mediated integration into the 89E11 site (GFP-ss-cFos-UTR in the plasmid pWALIUM-10 vector, http://www.flyrnai.org). We also obtained control GFP reporters of the K10 3’UTR (GFP-K10UTR in the UASp vector) or the *Ftz* intron (GFP-Ftz-intron in the pWALIUM-10 vector; integration into the 89E11 site by PhiC31). We used the Gal4/UAS system to drive expression of the GFP reporters in somatic (by Tj:Gal4) cells. We found that the GFP-K10UTR and the GFP-Ftz-intron transgenes were more highly expressed than the GFP-c-Fos-UTR transgene in somatic cells, as determined by IF and WB (Fig 4A–4C). In contrast, the GFP-c-Fos-UTR transgene was not repressed in larval cells (Fig 4D). Reducing *piwi* expression in somatic cells by RNAi increased the expression of GFP-c-Fos-UTR in somatic cells by approximately 2-fold (Fig 4E, S8A and S8B Fig); *piwi* reduction by RNAi phenocopies *piwi*[1/2] mutant [5, 6, 44]. These results suggest that the c-Fos 3’UTR is sufficient to reduce gene expression in ovarian somatic cells, and that Piwi is required for this repression.

### The c-Fos 3′ UTR recruits Piwi

**Fig 5:**
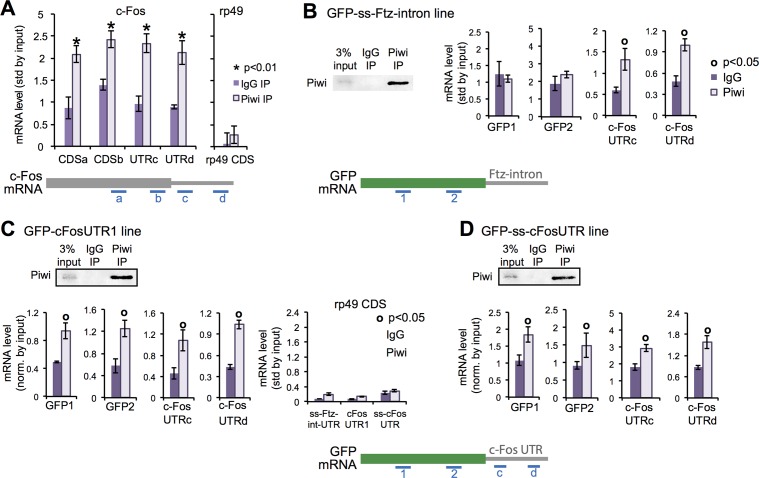
The 3′ UTR of c-Fos recruits Piwi. (A) RT-qPCR analysis of Piwi and IgG IP’d mRNAs. Diagram of the c-Fos mRNA indicates the regions amplified by primer sets a-d. (B) Western blot of Piwi and IgG IP from ovarian extract of Tj:GFP-ssFtz-UTR and RT-qPCR analysis of Piwi and IgG IP’d mRNAs. Diagram of the GFP mRNA indicates the regions amplified by primer sets 1 and 2. The 5% input served as normalization. (C) As in (B), using ovarian extract from Tj:GFP-c-Fos-UTR1 (generated by random integration). (D) as in (B), using Tj:GFP-ss-c-FosUTR (generated by site-specific integration). Error bars represent standard deviation, and the Student’s *t* test was used for statistical comparison.

Next, we examined whether Piwi protein interacts with the c-Fos transcripts. We found that the coding region and 3’UTR of c-Fos mRNA, but not rp49 mRNA, were enriched in a Piwi IP from wild-type ovarian cells compared with to IgG IP (Fig 5A). rp49 is a ribosomal subunit and expressed in the same cell types as c-Fos. This enrichment was also observed by crosslinking followed by IP (S8C Fig). To determine whether the c-Fos 3ʹ UTR is sufficient to recruit Piwi, we evaluated the enrichment of the GFP-Ftz-intron or GFP-c-Fos-UTR reporter mRNAs (both driven by Tj:Gal4) in Piwi IPs from ovarian cells. We found that GFP-c-Fos-3’UTR mRNA and endogenous c-Fos mRNA, but not GFP-Ftz-intron mRNA or rp49 mRNA (lacking the c-Fos 3’UTR), were enriched in Piwi IPs from ovarian cells (Fig 5B–5D). Thus, the c-Fos 3’UTR is sufficient to recruit Piwi.

### Gene repression induced by the c-Fos 3′ UTR coincides with increased piRNA production

**Fig 6:**
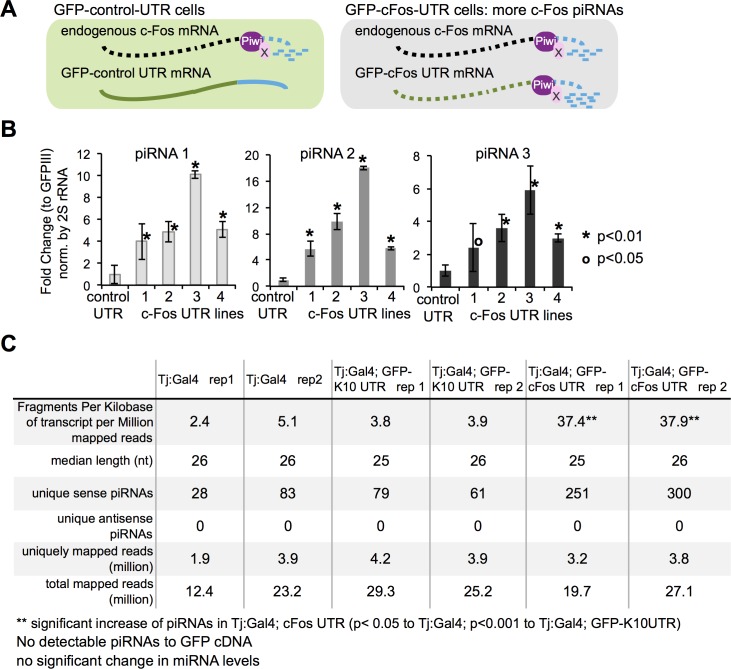
Gene repression mediated by the c-Fos 3′ UTR coincides with increased primary c-Fos-specific piRNAs. (A) Model of primary piRNA generation as a mode of gene repression predicts that the GFP-c-Fos-UTR will generate additional c-Fos piRNAs. (B) TaqMan RT-qPCR quantitation of piRNAs 1–3 unique to the *c-Fos* 3′ UTR from GFP-K10 UTR and 4 lines of GFP-c-Fos UTR. The 2S rRNA served as normalization. Error bars represent standard deviation, and the Student’s *t* test was used for statistical comparison. (C) piRNA profiling by Illumina sequencing of 2 biological replicate samples from (i) Tj:Gal4, (ii) Tj:Gal4; GFP-K10UTR, or (iii) Tj:Gal4; GFP-c-Fos UTR. Error bars represent standard deviation, and one-sided Student’s *t* test was performed for statistical analysis.

If Piwi and the c-Fos 3’ UTR repress gene expression through the generation of primary piRNAs, then the GFP-c-Fos-UTR transgene would be predicted to increase the biogenesis of these specific piRNAs (Fig 6A). We quantified the levels of c-Fos piRNAs 1–3 and found that they increase by 2- to 20-fold in GFP-c-Fos-3’UTR lines compared to control lines not expressing the transgene (Fig 6B). We then purified small RNAs from ovarian cells of Tj:Gal4, Tj:Gal4;GFP-K10UTR, or Tj:Gal4;GFP-c-Fos-3’UTR and performed RNA-seq. Computational filtering (see Materials and Methods) to identify piRNA sequences aligned to the c-Fos 3’UTR that were all in the sense orientation, had a median size of 26 nt, and contained the molecular signature of the first base being uridine in more than 70% of the piRNAs (Fig 6C and S8D Fig). The c-Fos-specific piRNAs increased by approximately 9 fold (37 versus 4 fragments per kilobase of transcript per million mapped reads in controls) and unique sense piRNA sequences increased by approximately 4–5 fold in in GFP-c-Fos-3’UTR ovarian tissue (Fig 6C). We did not detect antisense piRNAs unique to the c-Fos-3’UTR, increase in miRNAs, or piRNAs aligned to the GFP coding sequence. Thus, the repression of c-Fos and GFP-c-Fos-3’UTR coincides with increased primary piRNA generation by Piwi.

### c-Fos repression in ovarian somatic cells is required for female fecundity

**Fig 7:**
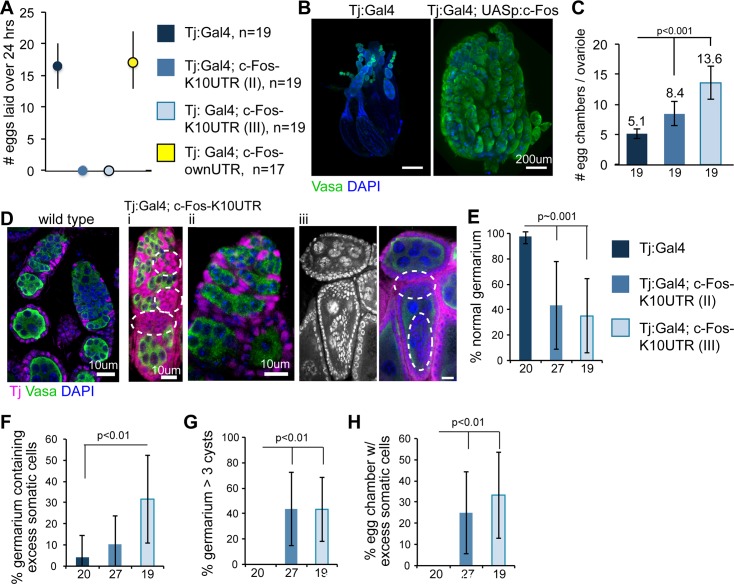
Ectopic expression of *c-Fos* in somatic stem cells and follicle cells results in animal infertility, ovarian tissue dysmorphogenesis, and somatic cell disorganization. (A) Quantitation of eggs laid by animals with Tj:Gal4 driving control, c-Fos CDS with *K10* UTR or c-Fos UTR. Median and twenty-fifth and seventy-fifth percentiles are indicated by circles and lines, respectively. *n* = sample size. (B) Vasa (green) IF and DAPI (blue) staining of ovaries, imaged by light sheet microscopy (C) Average number of egg chambers per ovariole. (D) Tj (magenta) and Vasa (green) IF and DAPI (blue) staining of germarium and egg chambers from wild-type or Tj:Gal4; c-Fos-K10 UTR. (i) Excessive and disorganized somatic cells in the germarium, (ii) germ-cell cyst accumulation in the germarium, and (iii) excessive and disorganized somatic cells that invade into the germ-cell compartment in the egg chambers are phenotypes observed in Tj:Gal4; c-Fos-K10 UTR. Dashed ovals indicate somatic cell disorganization or invasion. Quantitation of ovaries with (E) normal structure and organization, (F) excessive and disorganized somatic cells in the germarium, (G) >3 cysts in the germarium, and (H) egg chambers with excessive and disorganized somatic cells. In each graph, genotypes (color-coded as black, dark blue, and light blue) on panel E apply to F, G, and H, and numbers below the x-axis indicate the sample sizes. Error bars represent standard deviation. The Student’s *t* test was used for statistical analyses.

Given the developmental importance of *c-Fos* [19–21] and our finding that Piwi mediates *c-Fos* repression, we examined the functional consequences of ectopic *c-Fos* expression in the *Drosophila* ovary. To overexpress *c-Fos*, we replaced the 3’UTR in the *c-Fos* transgene with the K10 3’UTR to disrupt Piwi-mediated repression (S9A Fig). We also generated control animals with UASp:c-Fos containing its own 3′ UTR and a c-Fos knockdown line (c-Fos-*K10* 3’UTR; c-Fos shRNA-Val10). We found that overexpression of the *c-Fos*-*K10* 3’UTR transgene in somatic stem cells and somatic follicle cells abolished egg production (Fig 7A and S9A Fig). Further, egg production was restored in flies expressing c-Fos shRNA-Val10 to reduce *c-Fos-K10* 3’UTR in somatic stem cells and somatic follicle cells (S9B and S9C Fig). This finding showed that repression of *c-Fos* in ovarian somatic cells, mediated by its 3′ UTR, is required for female fecundity.

### *c-Fos* repression is required for ovarian tissue morphogenesis

Overexpression of somatic *c-Fos* by Tj:Gal4 driving c-Fos-K10UTR resulted in enlarged ovarian tissues, longer ovarioles, and more egg chambers per ovariole (Figs 7B and 7C and S9D). Vasa IF analysis showed that Vasa expression was persistent in all egg chambers with somatic *c-Fos* overexpression, but low in the mid- and late-stage egg chambers of control (Tj:Gal4) ovaries (Fig 7B). We observed defective egg chamber morphology in ovarioles overexpressing somatic *c-Fos* (S9E Fig) and rampant necrosis in late-stage germ cells (S9F Fig). These findings suggest that *c-Fos* overexpression in ovarian somatic cells results in the arrest of oocyte maturation, retention of egg chambers in the ovarioles, necrosis of late-stage germ cells, and failure of egg production by the animal.

Further analyses of germaria and egg chambers by Tj and Vasa IF staining revealed various cellular defects due to the overexpression of somatic *c-Fos* (Fig 7D), such as abnormal cell organization in 57%–65% of germaria (Fig 7E), excessive and disorganized somatic cells in 10% –32% of germaria (Fig 7Di and 7F), and cyst accumulation (>3 cysts) in 44% of germaria (Fig 7Dii and 7G). In 25%–33% of the egg chambers, *c-Fos* overexpression in somatic stem cells and somatic follicle cells led to disorganization, accumulation into multiple cell layers, and invasion into the germ cell compartment (Fig 7Diii and 7H). These findings indicate that *c-Fos* regulation is required for somatic stem cell and somatic cell organization for ovarian tissue morphogenesis. Quantitation of the S phase (IF of PCNA) and mitosis (IF of phosphorylated serine 10 in histone H3) revealed no differences between control cells and cells overexpressing *c-Fos* (S9G Fig). Therefore, overexpression of *c-Fos* does not increase cell proliferation in ovarian somatic stem cells and somatic cells.

**Fig 8:**
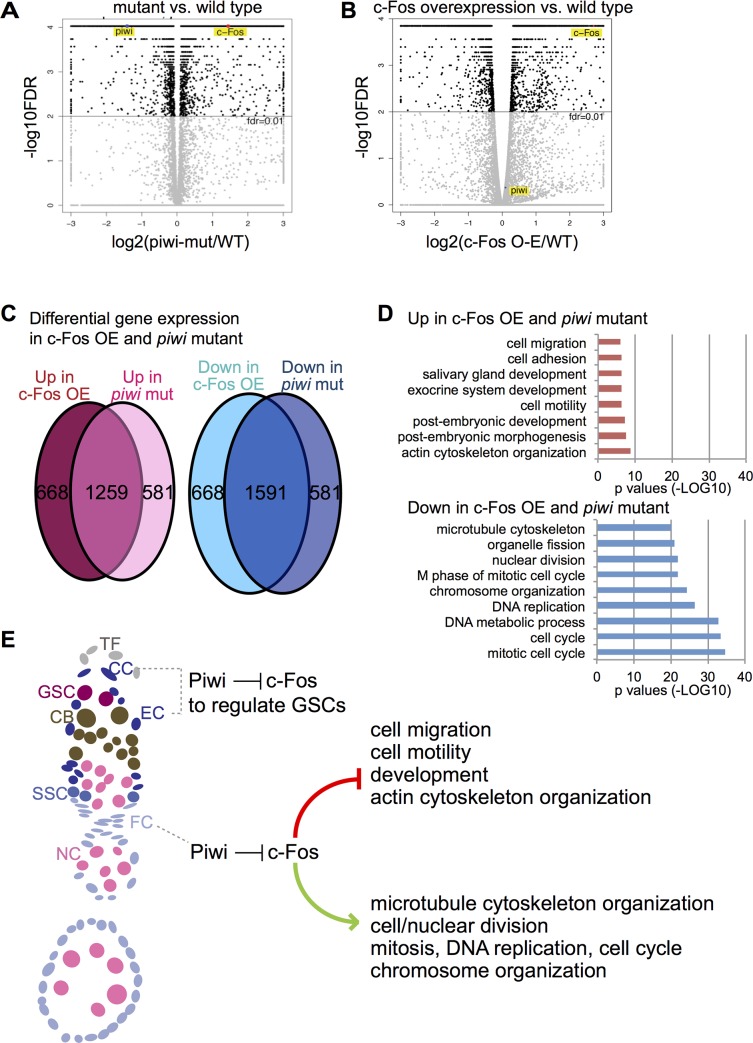
The transcriptomes are similar between the *piwi* mutant and somatic *c-Fos* overexpressing ovarian cells. Comparison of Gene expression profiles between (A) *piwi* mutant and wild type or (B) *c-Fos* overexpression and wild type by RNA-seq. c-Fos and Piwi are indicated in the comparative graphs. Upregulation and downregulation were determined by using a false discovery rate <0.01 and > 1.5-fold. Note that c-Fos is upregulated in both (A) and (B). (C) Venn diagrams indicate the overlap of upregulated and downregulated genes (compared to wild type) in *c-Fos* overexpression and *piwi* mutant ovarian cells. (D) Graphs indicate gene ontology categories and *p*-values of upregulated and downregulated genes in both c-Fos overexpression and *piwi* mutant. (E) Proposed model of c-Fos repression by Piwi in the somatic niche (cap cells and escort cells) to influence GSCs and in the somatic/follicle cells to regulate defined cellular processes. Labeled cell types are, respectively, TF, terminal filament; CC, cap cell; GSC, germline stem cell; CB, cystoblast; EC, escort cell; SSC, somatic stem cell; FC, follicle cell; NC, nurse cell; oocyte.

Our findings suggest that an important function of Piwi in the *Drosophila* ovary is to repress *c-Fos* in the somatic niche and somatic ovarian cells, and that animals with *piwi* loss of function and *c-Fos* overexpression share similar phenotypes, including somatic cell disorganization. Although we observed GSC loss or differentiation defects in *piwi* mutant flies but not *c-Fos*-overexpressing flies, this difference is likely due to the presence of Piwi in *c-Fos-*overexpressing ovaries. To examine the potential molecular similarities between animals with *piwi* loss and *c-Fos* overexpression, we compared the transcriptomes of the *piwi*[1/2] mutant and *c-Fos* overexpressing ovaries. In the gene expression profiling by RNA-seq, we found the mean FPKM values of c-Fos to be 12.6 in *w*[1118], 34.2 in *piwi*[1/2], and 70.1 in c-Fos-K10UTR (overexpressing transgenic c-Fos) ovarian cells (summarized in S7A). Remarkably, more than 65% of differentially expressed genes (compared to the wild type) were the same in the *piwi* mutant and *c-Fos* overexpressing ovaries (Fig 8A–8C). Genes upregulated by *c-Fos* overexpression or *piwi* loss were enriched in the functional categories of actin cytoskeleton organization, morphogenesis, development, and cell motility categories, whereas downregulated genes were enriched in microtubule cytoskeleton organization, cell cycle, cell division, mitosis, DNA replication, and chromosome organization (Fig 8D). We thus propose that Piwi regulates these processes by repressing *c-Fos* in the ovarian somatic cells (Fig 8E) to promote cell organization and tissue morphogenesis.

## Discussion

**Fig 9:**
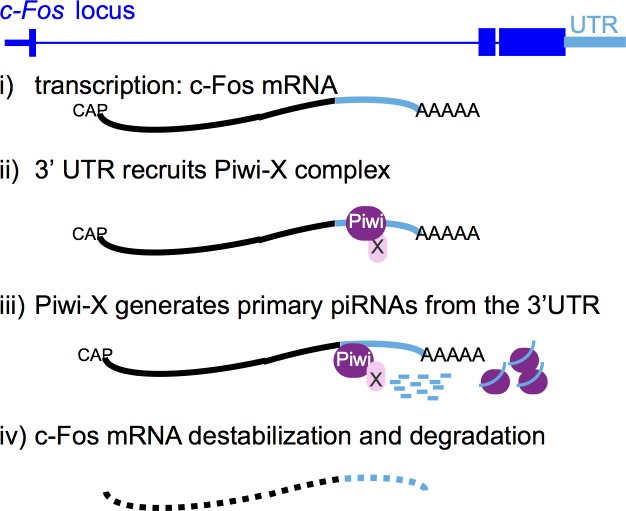
Proposed mechanism by which Piwi mediates c-Fos repression. Piwi protein and unknown associated factors are recruited by the c-Fos 3’UTR to bind the mRNA. The Piwi protein complex includes the 5’ and 3’ nucleases that generate the mature primary piRNAs. Processing of the c-Fos 3’UTR into mature piRNAs cause instability and degradation of the whole mRNA.

Piwi functions in both the somatic niche and in GSCs to maintain GSCs, but its underlying mechanisms are not well understood [4, 45]. We found that an important function of *piwi* in the *Drosophila* ovary development is to repress *c-Fos*. Piwi-mediated repression of c-Fos in somatic stem cells and somatic follicle cells was required for somatic cell organization and ovarian tissue morphogenesis. Our data suggest that the 3′ UTR of the c-Fos mRNA recruits Piwi, which regulates the activities of as yet identified nucleases to generate primary piRNAs from the c-Fos 3’UTR, leading to destabilization and post-transcriptional repression of *c-Fos* (Fig 9). Unclear aspects of the proposed model (Fig 9) include the mechanism by which the c-Fos 3’UTR recruits Piwi protein, identity of the nucleases involved in generating the primary piRNAs, and the extent by which the mRNA degradation machinery is involved. In GSCs, c-Fos expression was comparatively high and important for fertility, suggesting that a Piwi-independent mechanism regulates c-Fos.

Relatively little is known about how Piwi protein targets non-transposon mRNAs. Two recent genomic studies uncovered that piRNAs and the mouse Piwi protein MIWI cause instability of a subset of mRNAs in the mouse testes [46, 47]. Moreover, MIWI-mediated targeting of mRNAs and long noncoding RNAs depends on retrotransposon sequences and occurs in the cytoplasm [46]. Our study showed that the piRNAs need not be of retrotranspon origin (none of the piRNAs from the c-Fos 3’UTR are homologous to retrotransposon sequences) and that this gene regulation functionally impacts germ cell development and animal fertility, thereby contributing to the understanding of gene regulation by Piwi and piRNAs. Open questions include how Piwi and piRNAs target individual mRNAs, and whether a direct mechanism links Piwi-piRNAs and the mRNA degradation machinery to mediate gene repression.

The regulation of c-Fos and potentially other genes by Piwi-dependent processing into piRNAs in the ovary supports the concept that modest gene regulation is important during developmental events. Piwi and piRNAs repress the expression of *c-Fos* (a proto-oncogene with a pervasive role in development and disease) by an average of 2-fold. This modest repression is similar to that seen in the dosage compensation of sex chromosomes [48] or gene modulation by miRNAs [49]. However, the deregulation of these molecular processes can have severe, and often lethal, consequences on the developing organism. It is reasonable to propose that modest gene regulation by various molecular processes offers flexible modes of gene expression and potentially accommodates the many dynamic cellular events occurring during development.

Unexpectedly, c-Fos overexpression in the somatic niche did not significantly affect GSCs. This milder phenotype is likely a consequence of the nonoverlapping functions of Piwi and c-Fos in the somatic niche, and the regulation of additional molecular events by Piwi (e.g., dpp/BMP signaling) besides inhibiting *c-Fos* to affect GSC functions. Therefore, *c-Fos* appears to be a part of an extensive Piwi-centric network that safeguards GSC functions.

Our study uncovers a novel mechanism involving Piwi and c-Fos that regulates somatic cell organization for tissue morphogenesis of the *Drosophila* ovary. *piwi* reduction in the inner sheath cells or escort cells of ovaries is known to trigger somatic cell disorganization in the ovarioles [6]. This phenotype had not been studied in detail, likely because the tissue dysmorphogenesis phenotypes are masked by GSC loss and differentiation defects that occur in animals with mutations of *piwi* or factors in primary piRNA biogenesis [3, 4, 50]. Further, phenotypic analyses of *piwi* mutant mosaic clones in late-stage egg chambers revealed no observable defects [3, 4]. This finding indicates that *piwi* inactivation does not affect somatic follicle cells. However, the aforementioned somatic clonal analysis was carried out in differentiated follicle cells and not somatic stem cells, because *piwi* inactivation leads to loss of somatic stem cells. Our study circumvented this technical hurdle to uncover a function for Piwi in oogenesis.

Another intriguing finding was that *c-Fos* overexpression in somatic stem cells and somatic follicle cells was sufficient to result in persistent Vasa expression and arrest in egg chamber maturation (Fig 5*B*). This finding suggests that either c-Fos repression in the somatic cells is required for normal germ cell maturation or that *c-Fos* overexpression disrupts a yet-unidentified soma-to-germ cell signaling event that is required for normal germ cell maturation. Thus, the somatic cell organization mediated by c-Fos is likely not only important for tissue morphogenesis but also critical for ensuring germ cell maturation.

Non-transposon gene regulation by Piwi and piRNAs is not well-understood, possibly because only a few of these gene targets have been characterized, which are Tj [51], Nanos in embryonic axis determination [52], and Masc in sex determination of the silkworm [53]. Although piRNAs are generated from many genic transcripts in *Drosophila* ovaries, this often does not lead to repression of the genic transcripts. Our study is only the beginning of a more comprehensive effort to uncover non-transposon gene regulatory functions of Piwi and piRNAs to affect germ cell development. Future discoveries of other piRNA precursors repressed by Piwi and piRNAs in the germ cells and detailed mechanistic studies would be necessary to determine whether this c-Fos regulatory mechanism by Piwi and piRNAs is a broader post-transcriptional gene regulatory process.

## Materials and Methods

### Fly stocks & genetic assays

Adult *Drosophila* flies at day 4 post-eclosion were used for all genetic assays to ensure approximately developmental equivalency. Germ cell to somatic cell ratios were similar between wild type and *piwi*[1/2] mutants, as shown by similar Vasa and Tj levels (S1A and S1B Fig). Wild type is w[1118]. Most strains are from BDSC: *piwi*[1]/CyO (#43319), *piwi*[2]/CyO (#43637), *piwi*[06843]/CyO (deletion of *scar* and *piwi;* #12225) [5], Df(2L)BSC145 (deletion of chr2:32C1 that includes the *piwi* locus; #9505), *c-Fos*[EY01644]/TM3 (#15077), *c-Fos* [EY08232]/TM3 (#16882), Tj:Gal4 (#50105) Nos:Gal4 (#25751), UAS:GFP RNAi (#35786), UAS:shc-Fos (pVALIUM10; #27722), and UAS:shPiwi (pVALIUM20; #33724 & #34866). UAS:shPiwi-22235 is from VDRC. UAS:c-Fos RNAi (II) is from D. Bohmann [27]. UAS:c-Fos (II) and (III) contain the K10 UTR and are from P. Emery [54].

### Antibodies

Antibody names, IF dilutions (unless otherwise stated), and sources are as follows: Mouse 1B1 anti-Hts, 1:50, DSHB; rabbit anti-pMad, 1:300, Abcam ab52903; rabbit anti-c-Fos [55], 1:50, S. Subhabrata; guinea pig anti-Piwi, 1:200 for IF and 6ug/IP, Peng lab; mouse anti-Piwi [56], 1:100 for WB, M. Siomi; mouse anti-Aub [56], 1:200, M. Siomi; rabbit anti-c-Fos, 1:1000 for WB, Abnova PAB8948; rabbit anti-GFP, 1:500 and 1:2000 for WB, Life Technologies A011122; chicken anti-GFP, 1:250 and 1:2000 for WB, Life Technologies A10262; guinea pig anti-Tj, 1:250, Peng lab; rabbit anti-Vasa, 1:200, Santa Cruz sc-30210; Alexa dye–conjugated donkey secondary antibodies, 1:500, Jackson ImmunoResearch.

### Buffers

PBS is 137mM NaCl, 2.7mM KCl, 10mM Na_2_HPO_4_, 1.8mM KH_2_PO_4_ pH 7.4.

HEPM is 25 mM HEPES pH 7.9, 10 mM EGTA, 60 mM PIPES, 2 mM MgCl_2_.

Buffer D is 300mM KCl, 20mM HEPES pH 7.9, 0.2mM EDTA, 0.1% TritonX-100, 25% glycerol, 1x protease inhibitors (Roche, 11873580001), 1mM DTT.

### RT-qPCR

RNA was purified with the GeneJET RNA purification kit (Thermo Scientific, K0732). The cDNA was generated from 200 ng of RNA by using the High-Capacity cDNA RT kit with random hexamer or oligo dT (Applied Biosystems, 4374966). To distinguish sense from antisense transcripts, gene-specific reverse transcription was performed using Superscript IV Reverse Transcriptase (Thermo Scientific, 18090050). qPCR in the iQ SYBR Green Supermix (Bio-Rad, 170–8880) was analyzed on a Bio-Rad CFX96 system. RT reactions were performed in triplicate for quantitation. rp49 and rpl40 are used for normalization because they are ribosomal subunits with ubiquitous expression. S1 Table lists the primer sequences.

### Cytoplasmic and nuclear fractionation

Dissected ovaries were homogenized, extracted in buffer A with 0.1% Triton-X-100 for 4 minutes on ice, and centrifuged to obtain the supernatant as the cytoplasmic fraction. The nuclear pellet was washed once in buffer A and then extracted in buffer D to obtain the nuclear fraction.

### Western blotting

Equal amounts of protein extracts (in buffer D) were separated by SDS-PAGE and transferred onto a nitrocellulose membrane (162–0115, Bio-Rad). Membranes were blocked by 2% bovine serum albumin (BSA) in HEPM, incubated in primary antibodies (diluted in 1% BSA, HEPM 0.1% Triton X-100) overnight at 4°C, washed in PBS 0.1% Triton X-100, incubated in IRdye-conjugated secondary antibodies, and imaged on an Odyssey Fc system (LI-COR). Signals were quantitated with the Image Studio software (LI-COR). The Student’s *t* test was used for statistical analyses.

### Immunofluorescence

*Drosophila* ovaries were fixed in 3% paraformaldehyde in PBS, permeabilized in PBS with 0.3% Triton X-100 overnight at 4°C, blocked with 2% normal donkey serum in HEPM, probed with primary antibodies in HEPM and 0.05% Triton X-100 overnight at 4°C, washed with PBST, probed with secondary antibodies, washed with PBST, stained with DAPI, washed with PBST, and mounted in ProLong Gold Antifade Mountant (Life Technologies, P36930).

### Image acquisition and quantitation

The egg chamber and spectrosome were quantitated on a Zeiss Axio Imager.M2. GSCs were quantitated on a Nikon C2. Images were acquired with a Zeiss LSM780, a Leica TCS SP5, or a Zeiss LightSheet Z.1. For IF quantitation, images were acquired with the same parameters and analyzed by the Zen Black software (Zeiss) to obtain signal density, which is the background-subtracted average intensity per μm^2^. The Student’s *t* test was used for statistical analyses.

### Chromatin immunoprecipitation

Ovarian cytoplasmic fractions were extracted and discarded. Nuclear pellets were fixed, washed, and sonicated in the lysis buffer by using the Bioruptor Pico (Diagenode). Equal amounts of chromatin were added to Dynabeads (Life Technologies) prebound with 4ug of IgG or Piwi antibodies. After overnight incubation, beads were washed and immunoprecipitates were eluted. Purified DNAs from the input and eluates were analyzed by qPCR. S2 Table lists the primer sequences.

### piRNA reads analysis

Published piRNA sequences [12, 37–39] were downloaded from Gene Expression Omnibus (GEO; GSM154618, GSM154620, GSM154621, GSM154622, GSE9138, GSE13081, and GSE26507) to generate 2.2 million unique and 4.2 million multi-aligned libraries. For piRNAs from ovarian somatic cell lines, data [57] were downloaded from GEO (GSM1119289) and mapped to the BDGP R5/dm3 reference genome by using GSNAP [58]. 6.7 million reads were uniquely mapped. For in-house piRNA analysis, small RNAs were isolated by the mirVana miRNA isolation kit (Life Technologies, AM1561) and separated by PAGE gel. 50 ng of the gel-extracted RNAs (20–30 nt) was used to construct libraries with the TruSeq small RNA prep kit (Illumina, RS-200-0012). Libraries were sequenced on a Hi-Seq 2500 (Illumina). The adaptor-trimmed sequencing reads were aligned to the BDGP R5/dm3 reference genome by using GSNAP [58] and filtered by size and non-coding RNA type (tRNA, snoRNA, snRNA, rRNA, pre-miRNA, and miRNA). For miRNA quantification, reads were aligned to the miRBase hairpin precursors. The binomial test was used to compare piRNA enrichment at the c-Fos 3′ UTR against the rest of the c-Fos locus. The one-sided Student’s *t* test was performed to compare unique piRNAs. Seq data from this study were deposited in GEO by the identifier GSE69722. For analysis of small RNA data from Handler et al., data were downloaded from GEO (GSM1119289) and mapped to the BDGP R5/dm3 reference genome using GSNAP [58]. 6.7 million reads were uniquely mapped. Among them, 4751 were mapped to c-Fos (3R:25591717–25619835), 3660 of which mapped to the 3′ UTR (3R:25618747–25619835).

### smRNA isolation and TaqMan RT-qPCR

*Drosophila* ovaries were dissected and homogenized, and small RNAs were isolated by the mirVana miRNA isolation kit (Life Technologies, AM1561). smRNAs (5 ng each) were used in RT reactions with the TaqMan MicroRNA Reverse-Transcription Kit (Life Technologies, 4366596), and RT reactions were analyzed by the TaqMan 2S rRNA assay (Life Technologies, 4427975) and the custom smRNA assays 1–4 (Life Technologies, assays ID CS1RULS, CS20SR0, CS39QX8, and CSS07ER) by using TaqMan Universal MM II (Life Technologies, 4440043).

### RNAseq analysis of ovarian somatic stem cells (OSCs, Ohtani *et al*., 2013) and Gene Ontology analysis

To understand the unique interaction of piwi and c-Fos in ovary germline, we compared gene expression profiles between ovary germline and OSCs. PolyA-selected RNAseq data of OSC from Ohtani et al (2013) were downloaded from GEO (GSE47006) and mapped to the BDGP R5/dm3 reference genome using STAR [59], which was used in mapping of in-house generated RNAseq data. Gene expression values were estimated with Cufflinks [60], and compared with Cufflinks-generated expression of in-house data. Unsupervised hierarchical clustering analysis was done using all the genes that expressed (FPKM>1) in at least one samples. Differentially expressed genes were selected with p-value of less than 10^−5^ and fold change of greater than 4. Gene Ontology analysis was done using DAVID [61]. The biological difference between OSC and germline can be confounded by difference in data generation, however, gene ontology analysis of differentially expressed genes points to developmentally meaningful processes, thus indicating that the effect of biological differences is much stronger than the batch effect.

### Generation of transgenic *Drosophila*

The 3′ UTR was cloned into the pPGW vector (1077, *Drosophila* Genomics Resource Center) by Gateway (Life Technologies). Site-specific integration transgenic constructs were assembled by Gibson assembly and recombined into the pWALIUM10roe vector (TRiP, Harvard Medical School) by Gateway. Cesium chloride-prepped DNAs were sent to BestGene, Inc., for injection into w[1118] or Bloomington stock 9744 (integration site at 89E11) embryos to generate transgenic lines.

## Supporting Information

S1 Fig Molecular and cellular phenotypes of *piwi* reduction or *piwi* and *c-Fos* reduction in the *Drosophila* ovarian somatic cells. (A) RT-qPCR quantitation of Vasa, Tj, and Gapdh (standardized by rp49) mRNAs in ovarian cells from the wild type and *piwi*[1/2] mutant. (B) WB quantitation of Vasa, Tj, and Gapdh (standardized by the Coomassie staining signal) proteins in ovarian cells from the wild type and *piwi*[1/2] mutant. The top portion of the gel was Coomassie stained to show loading. (C) Quantification of Drosophila females with large (partially suppressed ovariole defects as shown in 1Biii) ovaries in piwi[1/1], c-Fos/+;piwi[1/1], piwi[2/06843], and c-Fos/+;piwi[2/06843]. (D) Percentage of eclosed adults that are *c-Fos* [EY08232] or actin:Gal4/*c-Fos*OE; *c-Fos*[EY08232]. (E) c-Fos and actin WB of ovarian extract from animals with Nos:Gal4 driving GFP shRNA or c-Fos shRNA III. (F) c-Fos IF staining of ovaries from Nos:Gal4 driving control shRNA and *c-Fos* shRNAs II and Val10. (G) Quantification of Drosophila females with large (partially suppressed ovariole defects as shown in 1Biii) ovaries in animals with Nos:Gal4 (germ cell-specific) driving Piwi and c-Fos shRNAs. (H) Quantification of Drosophila females with large (partially suppressed ovariole defects as shown in 1Biii) ovaries in animals with Tj:Gal4 (somatic cell-specific) driving Piwi and c-Fos shRNAs. Error bars represent standard deviations, and the Student’s *t* test was used for statistical comparison.
(PDF)

S2 Fig *c-Fos* in the germ cells is required for animal fertility. (A) The number of eggs laid by animals with Nos:Gal4 driving control or c-Fos shRNAs during a 24-h period. The black square represents the median value, and the lines indicate the twenty-fifth to seventy-fifth percentiles. *n* = sample size. Average numbers of (B) GSCs per germarium, (C) PCNA-positive (indicating S phase) GSCs per ovary (D) PCNA-positive (indicating S phase) germ cells per ovary, and (E) phosphorylated serine10 of histone H3-positive (indicating mitosis) germ cells per ovary in animals with Nos:Gal4 driving control or c-Fos shRNA. Error bars represent standard deviations. Analysis of (F) Orb, (G) phosphorylated H2Av, (H) actin by phalloidin staining, and (I) Gurken in ovarioles with Tj:Gal4 driving control or c-Fos shRNA-Val10. Orb is used to analyze oocyte specification [62, 63]. Phosphorylated H2Av indicates meiotic double-stranded breaks [64]. Phalloidin stains actin, which makes up the ring canal structure that connects nurse cells and the oocyte. Gurken is used to analyze oocyte axis patterning [65].
(PDF)

S3 Fig *c-Fos* reduction partially rescue germline stem cell maintennace and differentiation. (A) Vasa (green) and Hts (magenta) IF and DAPI (blue) staining of germaria of the indicated genotype. (B) The average number of spectrosomes per germarium. (C) Quantification of germaria with 3 or more egg chambers. (D) The average number of egg chambers per ovariole. The *piwi* mutant alleles are 1, 2, and 06843, and *c-Fos* mutant alleles are EY01644 (01644) and EY08232 (08232). Error bars represent standard deviations, and the Student’s *t* test was used for statistical comparison.
(PDF)

S4 Fig Reduction of *c-Fos* does not impact dpp/BMP signaling or the JNK pathway. (A) Confocal images of pMad (phosphorylated Mad) and Hts IF in the germaria of (i) *piwi*[1/+], (ii) *piwi*[1/2], (iii) *piwi*[1/2]; *c-Fos*[EY08232/+], (iv) Tj:Gal4; piwi-shRNA 22235, (v) Tj:Gal4; piwi-shRNA 22235; c-Fos shRNA Val10 (III). White circles indicate pMad-positive germ cells. Bar, 10um. (B) Quantitation of pMad-positive cells in *piwi*[1/+], *piwi*[1/2], *piwi*[1/2]; *c-Fos*[EY08232/+], and *piwi*[1/2]; *c-Fos*[EY01644/+]. The numbers of pMad-positive cells in *piwi*[1/2], *piwi*[1/2]; *c-Fos*[EY08232/+], and *piwi*[1/2]; *c-Fos*[EY01644/+] are significantly more than that in *piwi*[1/+]. (C) Quantitation of pMad-positive cells in Tj:Gal4 driven control, piwi-shRNA22235, piwi-shRNA33724, piwi-shRNA22235; c-Fos shRNA Val10 (III), and piwi-shRNA33724; c-Fos shRNA (II). The numbers of pMad-positive cells in Tj:Gal4 driven piwi-shRNA22235, piwi-shRNA33724, piwi-shRNA22235; c-Fos shRNA Val10 (III) are significantly more than that in control. (D) Quantification of Drosophila females with large (partially suppressed ovariole defects as shown in 1Biii) ovaries in animals with Tj:Gal4 driving Piwi shRNA-22235, in addition to control (no shRNA), bsk-shRNA 36690, and bsk-shRNA 32977. The Student’s *t* test was used to calculate the p values.
(PDF)

S5 Fig *Piwi* mutations do not affect RNA polymerase II binding at the *c-Fos* locus. (A) RT-qPCR quantitation of RPL40 and c-Fos (normalized rp49) mRNAs in ovarian cells from the wild type and *ovo* mutant. Two primer pairs targeting c-Fos were used. Two primer sets targeting to the c-Fos mRNA were utilized to demonstrate consistency. Averages in RT-qPCR were of 3 RT reactions. (B) Chromatin immunoprecipitation of RNA polymerase II and IgG from wild-type and *piwi* mutant ovarian cells. c-Fos promoter, intergenic regions, rp49 promoter, and RPL40 promoter were assayed. Error bars represent standard deviations. The Student’s *t* test was used to for statistical analysis.
(PDF)

S6 Fig Detection of piRNAs from c-Fos UTR by TaqMan RT-qPCR. (A) piRNA gene targets were ranked by the read density (read number/bp of 3′ UTR) of uniquely mapped piRNAs. Some genes were highlighted for comparison to c-Fos, whose piRNAs were of relatively low abundance. (B) Schematic diagram of small RNA detection by TaqMan assays. A looped RT primer annealed to a piRNA is used for first-strand cDNA synthesis. Following second-strand synthesis, the TaqMan probe binds to both piRNA and RT primer sequence. The NFQ (non-fluorescent quencher) at the 3′ end of the probe quenches the FAM dye at the 5’ end. The MGB (minor groove binder) stabilizes probe binding. PCR primers specific to piRNA sequence and the looped RT primer allow for cycling PCR reaction that degrades the probe bound to the piRNA-RT primer junction. This degradation releases the FAM (from NFQ) to be able to fluoresce, and the FAM signals are quantitated as a readout of piRNA amount. Other small RNAs, such as 2S rRNA, can be also be quantitated by separate sets of probes and primers. The combination of the looped RT primer, the probe and PCR primers results in ~10,000-fold sensitivity to the mature small RNA than the precursor (Life Technologies). (C) The piRNAs unique to the 3′ UTR of c-Fos mRNA and targeted by TaqMan probes for RT-qPCR. (D) TaqMan RT-qPCR quantitation of piRNAs 1–3 in ovarian cells from Tj:Gal4 driving control or c-Fos shRNA-II. Asterisks indicate *p*<0.001 by the Student’s *t*-test. (E) Western blotting (top panel) of IgG and Piwi IP of ovarian extract indicates high enrichment of Piwi. The immunoprecipitated RNA were P32-end labeled and analyzed by urea-PAGE (bottom panel). Signals from radioactive P32 were captured by autoradiogram.
(PDF)

S7 Fig Analysis of RNA-seq in the *Drosophila* ovarian cells and OSC. (A) RNA-seq data of OSCs from two studies were obtained. FPKM values of c-Fos from Sienski et al. were calculated by the investigator’s in-house perl script, and FPKM values of c-Fos from Ohtani et al. were calculated by using Cufflinks. Levels of c-Fos expression in OSCs were high and unchanged by knockdown of piRNA biogenesis factors. Our RNA-seq analysis, in triplicates, of wild type, *piwi* mutant, and *c-Fos* overexpressing ovarian cells. The level of *c-Fos* in *piwi*/wt ratio is ~3.4-fold at FDR (false discovery rate) of 2.45x10^-5^. (B) Unsupervised gene expression clustering of all genes in OSCs and germline tissues. In OSCs, siRNAs of genes were indicated. Blue-red heat map is of the z-scores. (C) Gene ontology analysis of the germline vs. the OSCs. P values in–LOG_10_.
(PDF)

S8 Fig Analysis of GFP-c-FosUTR reporter and Piwi IP from crosslinked ovarian cells. (A) WB of Piwi and Coomassie staining of the lower portion of the gel to show the loading control of the ovarian cell extract from Piwi-shRNA22235 or GFP-shRNA. (B) Representative WB of GFP or α-tubulin in ovarian cells from Tj:Gal4; GFP-c-Fos UTR’ shRNA control or Tj:Gal4; Piwi-shRNA; GFP-c-Fos UTR, normalized to α-tubulin WB signals. (C) RT-qPCR of c-Fos and rp49 of IgG and Piwi IPs from crosslinked ovarian cells. (D) Quantitation of bases in the first 15 nt of sense and antisense piRNAs from (i) Tj:Gal4, (ii) Tj:Gal4;GFP-K10 UTR, or (iii) Tj:Gal4;GFP-c-Fos UTR. Approximately 70% of all first-position nucleotides in the isolated piRNAs were uridine in RNA/thymidine in the reverse-transcribed cDNA.
(PDF)

S9 Fig Analyses of ovaries with somatic *c-Fos* overexpression. c-Fos and α-tubulin WB of ovarian extract from animals with Tj:Gal4 driving (A) (i) control, (ii) c-Fos overexpression II or III, (iii) c-Fos-ownUTR, (B) (iv) control, (v) c-Fos overexpression II, or (vi) c-Fos overexpression II; c-Fos shRNA-Val10. (C) Number of eggs laid by animals with Tj:Gal4 driving (iv) control, (v) c-Fos overexpression II, or (vi) c-Fos overexpression II; c-Fos shRNA-Val10 during a 24-h period. The black square represents the median value, and the lines indicate the twenty-fifth to seventy-fifth percentiles. (D) Vasa (green) IF in ovaries of Tj:Gal4 driving c-Fos overexpression II. Bar, 100 μm. The numbers indicate the first and ninth egg chamber in the highlighted ovariole. (E) Tj (red) and Vasa (green) IF of ovaries from Tj:Gal4 driving control or c-Fos overexpression II. Bars, 50 μm and 100 μm. White circles indicate oocyte in the same stage of the egg chamber. (F) DAPI-stained ovarioles in Tj:Gal4 driving c-Fos overexpression III. In later stages, germ cells appear necrotic. (G) Average PCNA-positive (indicating S phase) or phosphorylated serine 10 in histone H3-positive (indicating mitosis) ovarian cells per germarium from animals of Tj:Gal4 driving control or c-Fos overexpression (III).
(PDF)

S1 Table Sequences of primers used for RT-qPCR. (PDF)

S2 Table Sequences of primers used for ChIP-qPCR. (PDF)

S1 Text Supplementary Text. (DOCX)
