# Supplementary figures and images for: c-Fos Repression by Piwi Regulates Drosophila Ovarian Germline Formation and Tissue Morphogenesis
Source: PLoS Genet. 2016 Sep 13;12(9):e1006281. doi: 10.1371/journal.pgen.1006281 (PMC5021354; doi:10.1371/journal.pgen.1006281)

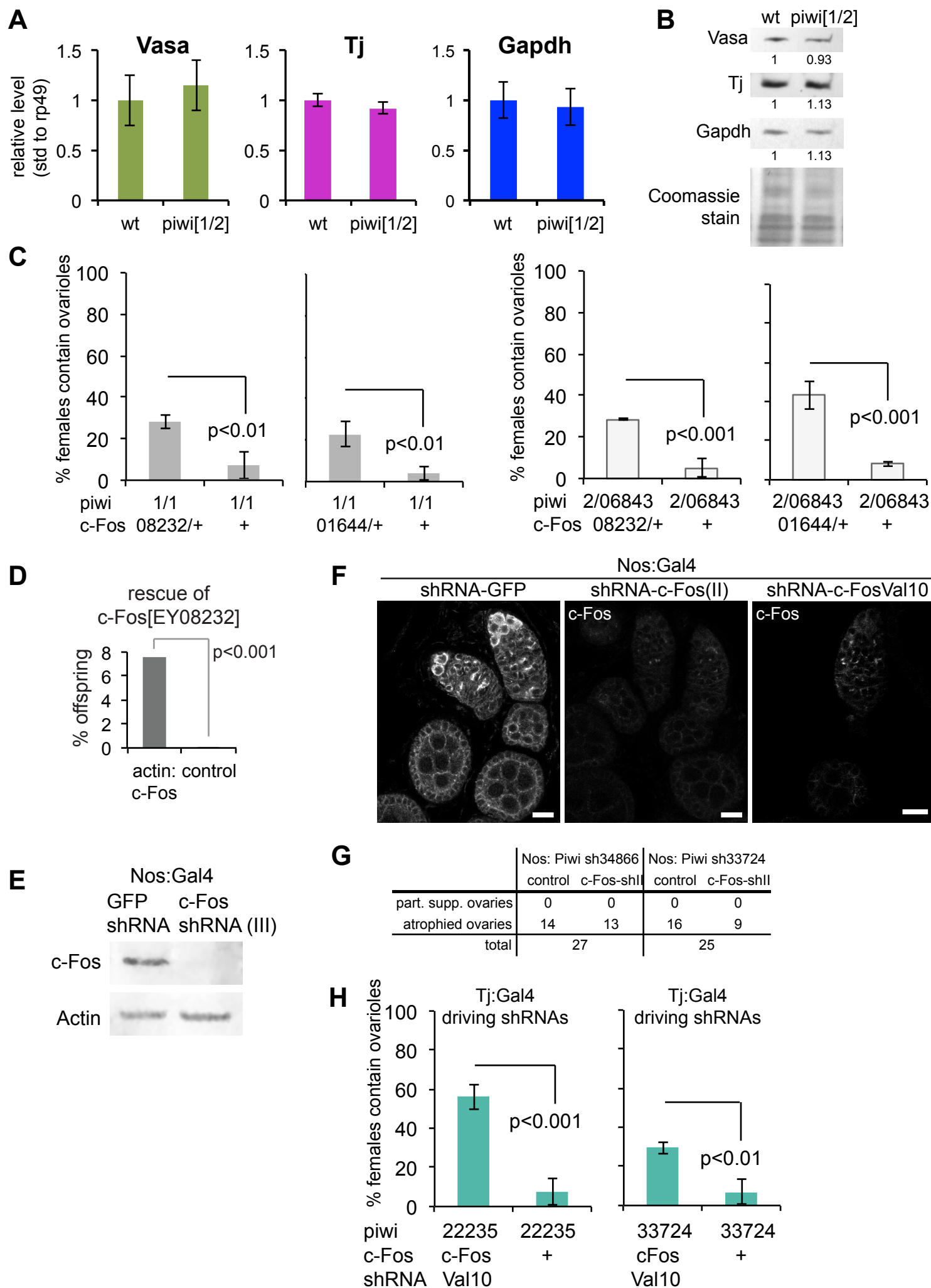

Supplement: S1 Fig — (A) RT-qPCR quantitation of Vasa, Tj, and Gapdh (standardized by rp49) mRNAs in ovarian cells from the wild type and piwi[1/2] mutant. (B) WB quantitation of Vasa, Tj, and Gapdh (standardized by the Coomassie staining signal) proteins in ovarian cells from the wild type and piwi[1/2] mutant. The top portion of the gel was Coomassie stained to show loading. (C) Quantification of Drosophila females with large (partially suppressed ovariole defects as shown in 1Biii) ovaries in piwi[1/1], c-Fos/+;piwi[1/1], piwi[2/06843], and c-Fos/+;piwi[2/06843]. (D) Percentage of eclosed adults that are c-Fos [EY08232] or actin:Gal4/c-FosOE; c-Fos[EY08232]. (E) c-Fos and actin WB of ovarian extract from animals with Nos:Gal4 driving GFP shRNA or c-Fos shRNA III. (F) c-Fos IF staining of ovaries from Nos:Gal4 driving control shRNA and c-Fos shRNAs II and Val10. (G) Quantification of Drosophila females with large (partially suppressed ovariole defects as shown in 1Biii) ovaries in animals with Nos:Gal4 (germ cell-specific) driving Piwi and c-Fos shRNAs. (H) Quantification of Drosophila females with large (partially suppressed ovariole defects as shown in 1Biii) ovaries in animals with Tj:Gal4 (somatic cell-specific) driving Piwi and c-Fos shRNAs. Error bars represent standard deviations, and the Student’s t test was used for statistical comparison. (PDF) [file pgen.1006281.s001.pdf]

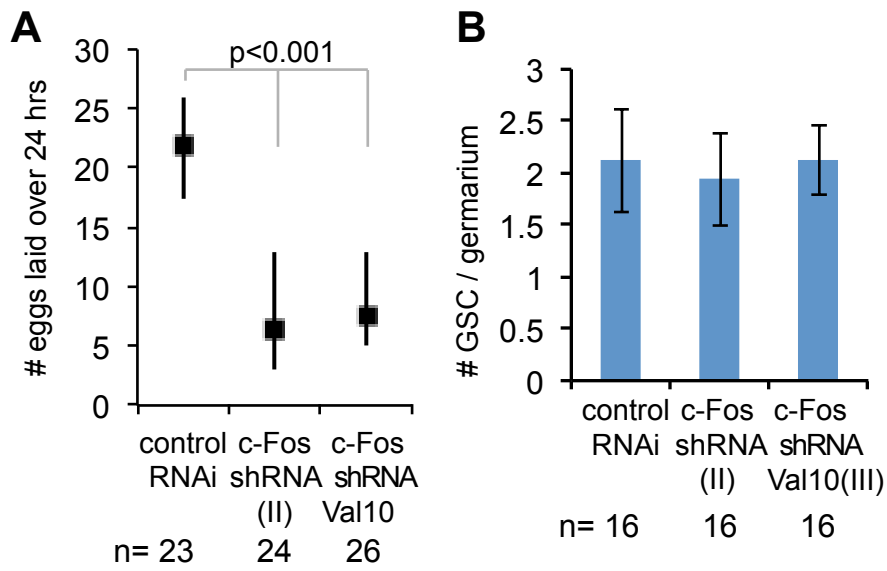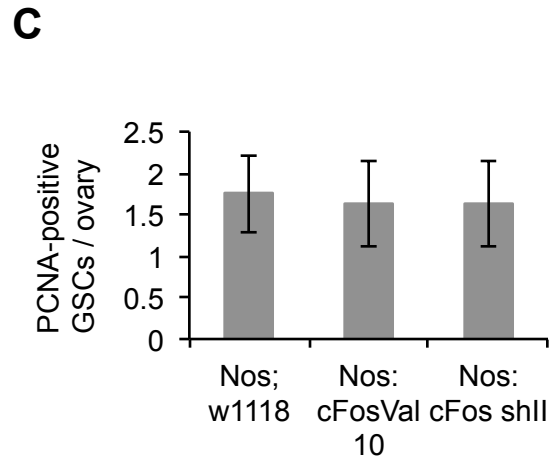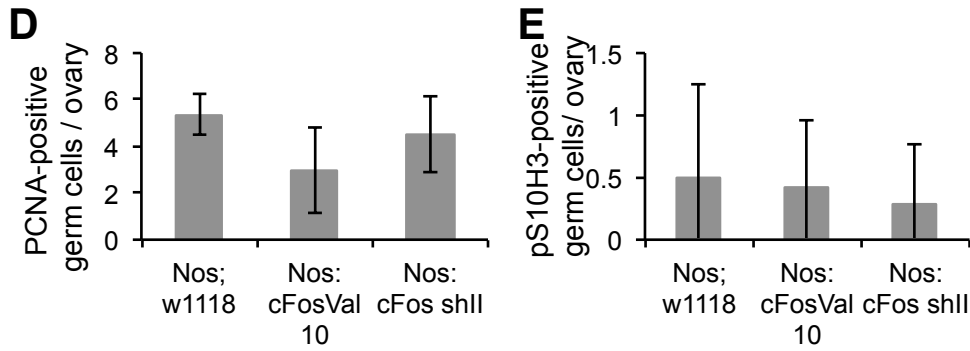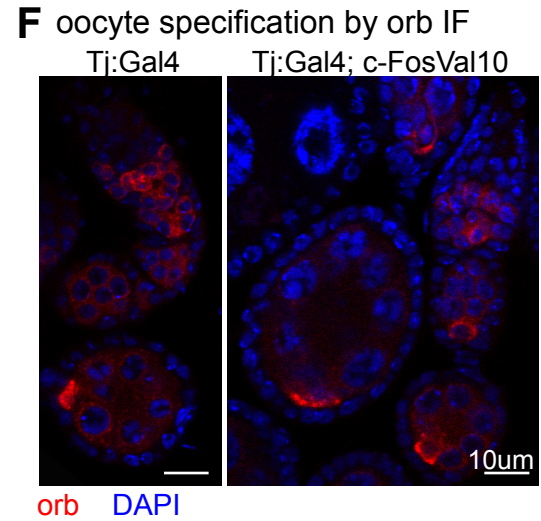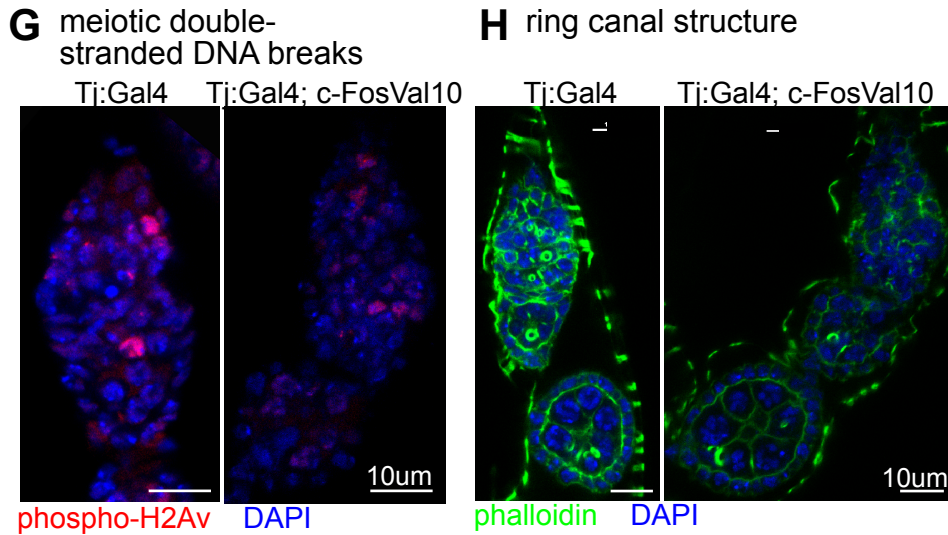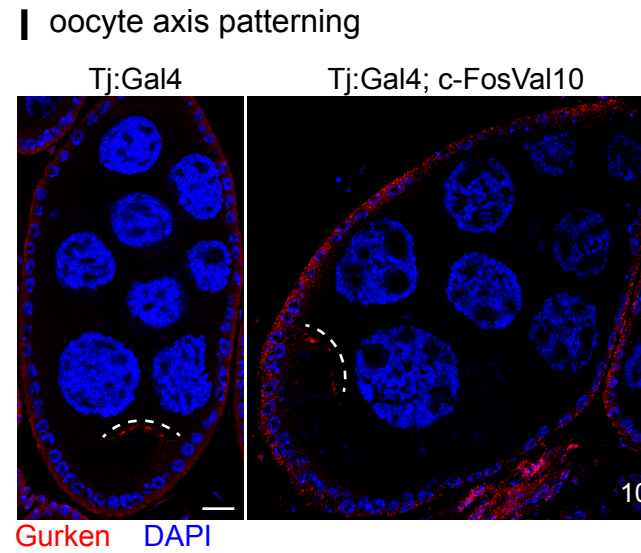

Supplement: S2 Fig — (A) The number of eggs laid by animals with Nos:Gal4 driving control or c-Fos shRNAs during a 24-h period. The black square represents the median value, and the lines indicate the twenty-fifth to seventy-fifth percentiles. n = sample size. Average numbers of (B) GSCs per germarium, (C) PCNA-positive (indicating S phase) GSCs per ovary (D) PCNA-positive (indicating S phase) germ cells per ovary, and (E) phosphorylated serine10 of histone H3-positive (indicating mitosis) germ cells per ovary in animals with Nos:Gal4 driving control or c-Fos shRNA. Error bars represent standard deviations. Analysis of (F) Orb, (G) phosphorylated H2Av, (H) actin by phalloidin staining, and (I) Gurken in ovarioles with Tj:Gal4 driving control or c-Fos shRNA-Val10. Orb is used to analyze oocyte specification [62, 63]. Phosphorylated H2Av indicates meiotic double-stranded breaks [64]. Phalloidin stains actin, which makes up the ring canal structure that connects nurse cells and the oocyte. Gurken is used to analyze oocyte axis patterning [65]. (PDF) [file pgen.1006281.s002.pdf]

**A**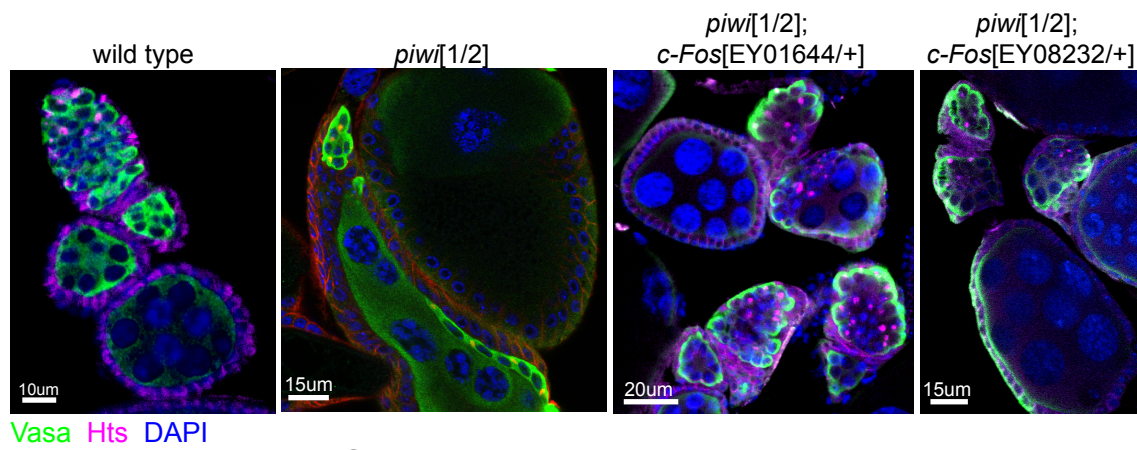

Vasa Hts DAPI

**B**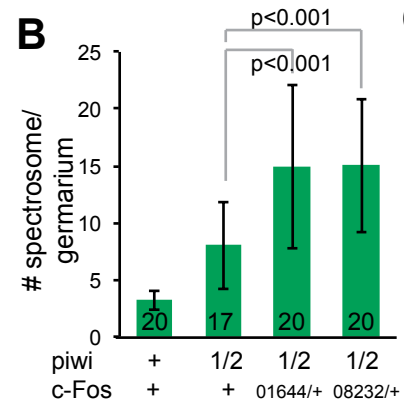**C**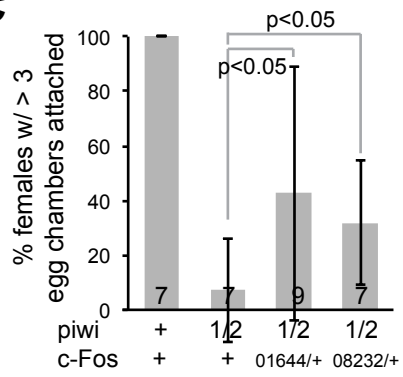**D**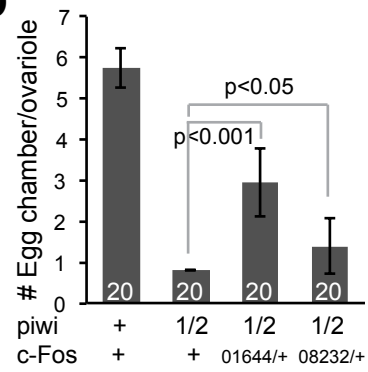

Supplement: S3 Fig — (A) Vasa (green) and Hts (magenta) IF and DAPI (blue) staining of germaria of the indicated genotype. (B) The average number of spectrosomes per germarium. (C) Quantification of germaria with 3 or more egg chambers. (D) The average number of egg chambers per ovariole. The piwi mutant alleles are 1, 2, and 06843, and c-Fos mutant alleles are EY01644 (01644) and EY08232 (08232). Error bars represent standard deviations, and the Student’s t test was used for statistical comparison. (PDF) [file pgen.1006281.s003.pdf]

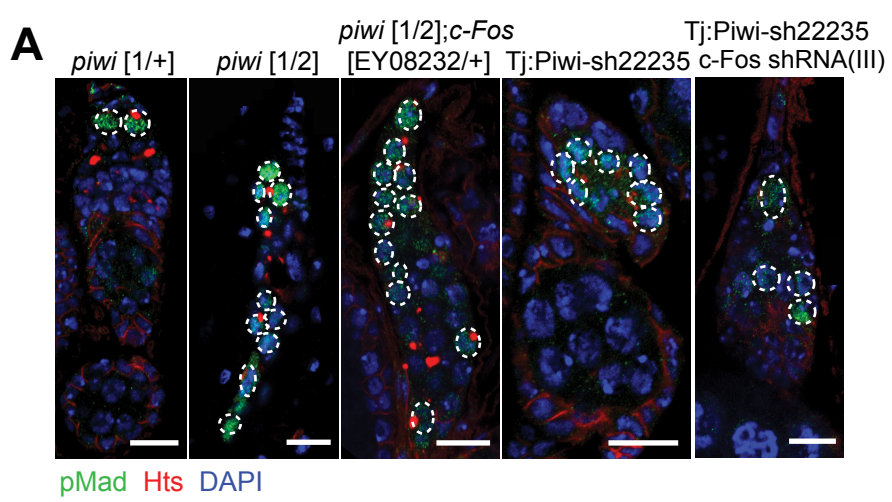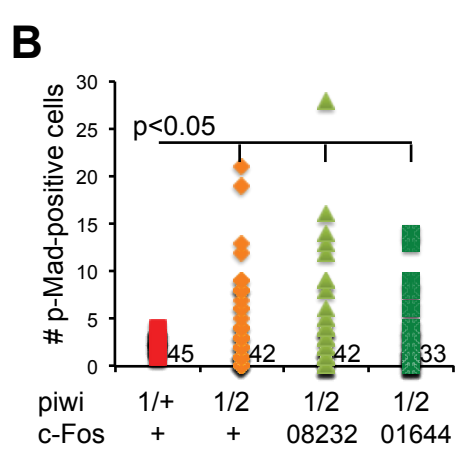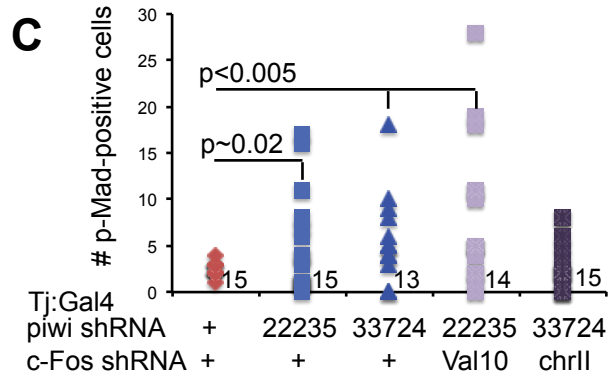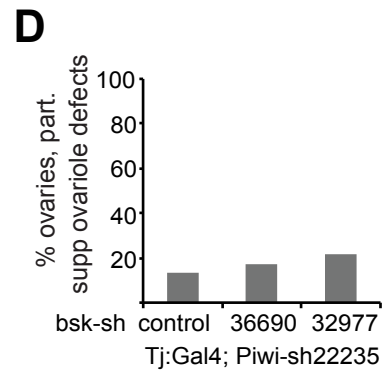

Supplement: S4 Fig — (A) Confocal images of pMad (phosphorylated Mad) and Hts IF in the germaria of (i) piwi[1/+], (ii) piwi[1/2], (iii) piwi[1/2]; c-Fos[EY08232/+], (iv) Tj:Gal4; piwi-shRNA 22235, (v) Tj:Gal4; piwi-shRNA 22235; c-Fos shRNA Val10 (III). White circles indicate pMad-positive germ cells. Bar, 10um. (B) Quantitation of pMad-positive cells in piwi[1/+], piwi[1/2], piwi[1/2]; c-Fos[EY08232/+], and piwi[1/2]; c-Fos[EY01644/+]. The numbers of pMad-positive cells in piwi[1/2], piwi[1/2]; c-Fos[EY08232/+], and piwi[1/2]; c-Fos[EY01644/+] are significantly more than that in piwi[1/+]. (C) Quantitation of pMad-positive cells in Tj:Gal4 driven control, piwi-shRNA22235, piwi-shRNA33724, piwi-shRNA22235; c-Fos shRNA Val10 (III), and piwi-shRNA33724; c-Fos shRNA (II). The numbers of pMad-positive cells in Tj:Gal4 driven piwi-shRNA22235, piwi-shRNA33724, piwi-shRNA22235; c-Fos shRNA Val10 (III) are significantly more than that in control. (D) Quantification of Drosophila females with large (partially suppressed ovariole defects as shown in 1Biii) ovaries in animals with Tj:Gal4 driving Piwi shRNA-22235, in addition to control (no shRNA), bsk-shRNA 36690, and bsk-shRNA 32977. The Student’s t test was used to calculate the p values. (PDF) [file pgen.1006281.s004.pdf]

**A**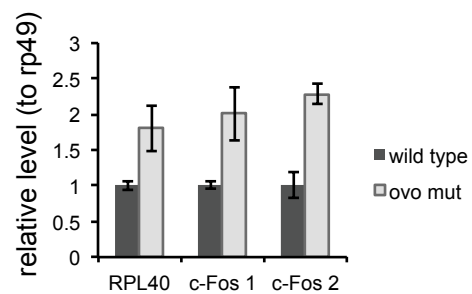**B**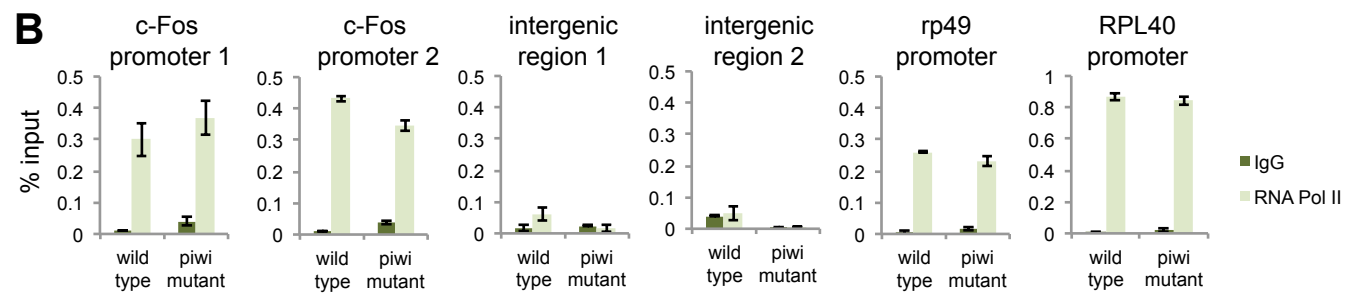

Supplement: S5 Fig — (A) RT-qPCR quantitation of RPL40 and c-Fos (normalized rp49) mRNAs in ovarian cells from the wild type and ovo mutant. Two primer pairs targeting c-Fos were used. Two primer sets targeting to the c-Fos mRNA were utilized to demonstrate consistency. Averages in RT-qPCR were of 3 RT reactions. (B) Chromatin immunoprecipitation of RNA polymerase II and IgG from wild-type and piwi mutant ovarian cells. c-Fos promoter, intergenic regions, rp49 promoter, and RPL40 promoter were assayed. Error bars represent standard deviations. The Student’s t test was used to for statistical analysis. (PDF) [file pgen.1006281.s005.pdf]

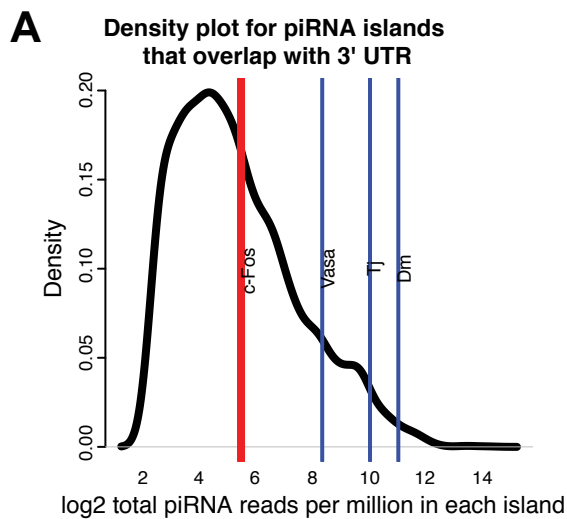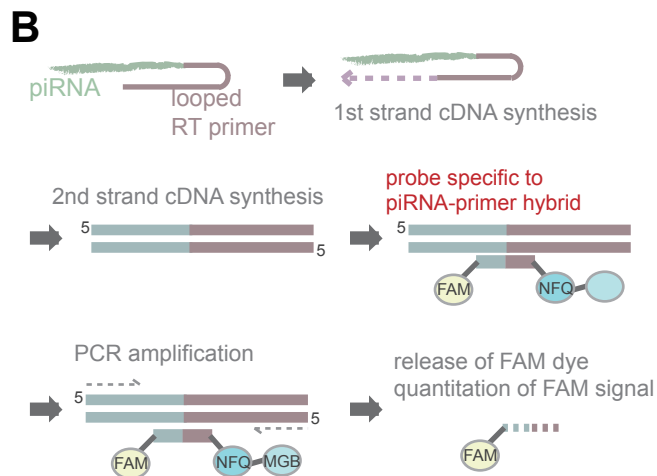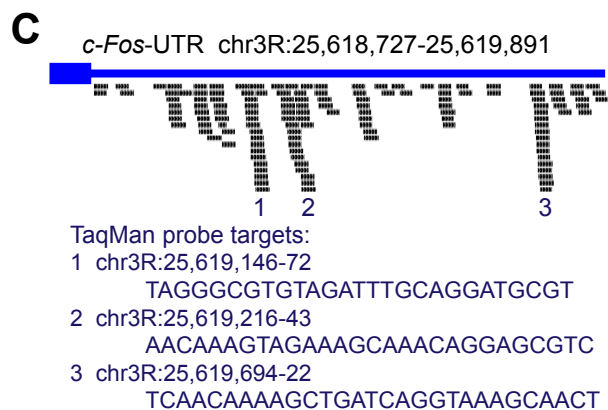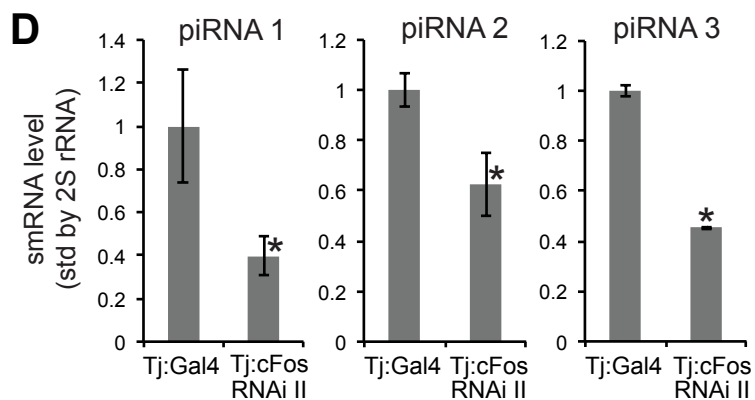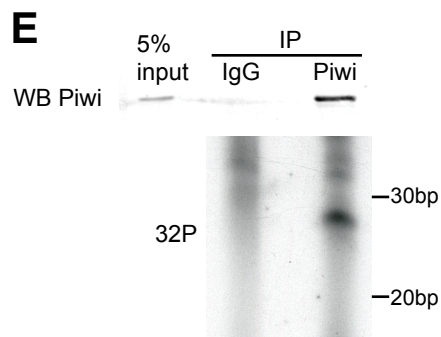

Supplement: S6 Fig — (A) piRNA gene targets were ranked by the read density (read number/bp of 3′ UTR) of uniquely mapped piRNAs. Some genes were highlighted for comparison to c-Fos, whose piRNAs were of relatively low abundance. (B) Schematic diagram of small RNA detection by TaqMan assays. A looped RT primer annealed to a piRNA is used for first-strand cDNA synthesis. Following second-strand synthesis, the TaqMan probe binds to both piRNA and RT primer sequence. The NFQ (non-fluorescent quencher) at the 3′ end of the probe quenches the FAM dye at the 5’ end. The MGB (minor groove binder) stabilizes probe binding. PCR primers specific to piRNA sequence and the looped RT primer allow for cycling PCR reaction that degrades the probe bound to the piRNA-RT primer junction. This degradation releases the FAM (from NFQ) to be able to fluoresce, and the FAM signals are quantitated as a readout of piRNA amount. Other small RNAs, such as 2S rRNA, can be also be quantitated by separate sets of probes and primers. The combination of the looped RT primer, the probe and PCR primers results in ~10,000-fold sensitivity to the mature small RNA than the precursor (Life Technologies). (C) The piRNAs unique to the 3′ UTR of c-Fos mRNA and targeted by TaqMan probes for RT-qPCR. (D) TaqMan RT-qPCR quantitation of piRNAs 1–3 in ovarian cells from Tj:Gal4 driving control or c-Fos shRNA-II. Asterisks indicate p<0.001 by the Student’s t-test. (E) Western blotting (top panel) of IgG and Piwi IP of ovarian extract indicates high enrichment of Piwi. The immunoprecipitated RNA were P32-end labeled and analyzed by urea-PAGE (bottom panel). Signals from radioactive P32 were captured by autoradiogram. (PDF) [file pgen.1006281.s006.pdf]

**A**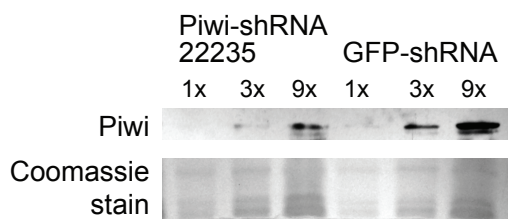**B**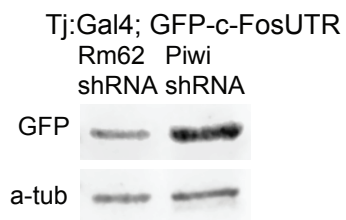**C**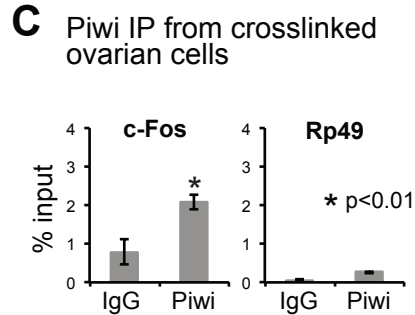**D**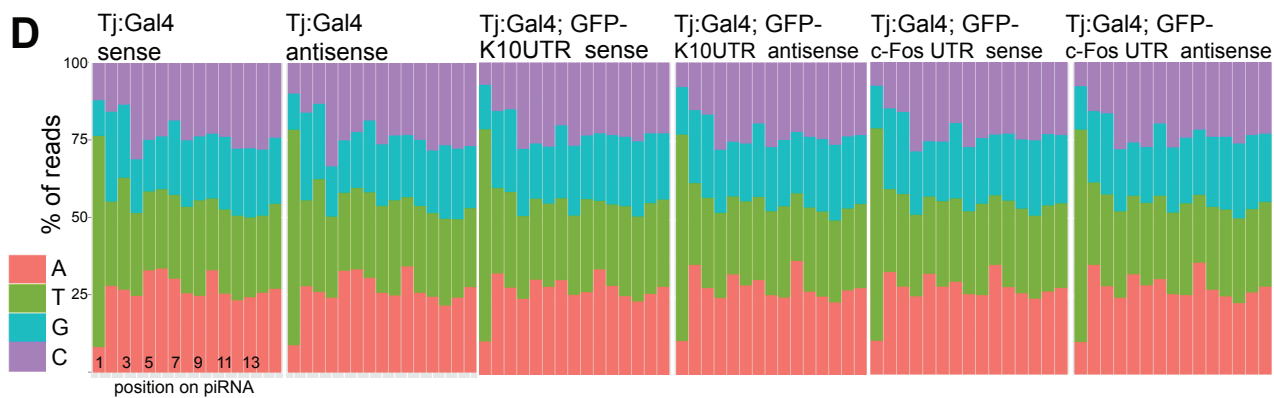

Supplement: S8 Fig — (A) WB of Piwi and Coomassie staining of the lower portion of the gel to show the loading control of the ovarian cell extract from Piwi-shRNA22235 or GFP-shRNA. (B) Representative WB of GFP or α-tubulin in ovarian cells from Tj:Gal4; GFP-c-Fos UTR’ shRNA control or Tj:Gal4; Piwi-shRNA; GFP-c-Fos UTR, normalized to α-tubulin WB signals. (C) RT-qPCR of c-Fos and rp49 of IgG and Piwi IPs from crosslinked ovarian cells. (D) Quantitation of bases in the first 15 nt of sense and antisense piRNAs from (i) Tj:Gal4, (ii) Tj:Gal4;GFP-K10 UTR, or (iii) Tj:Gal4;GFP-c-Fos UTR. Approximately 70% of all first-position nucleotides in the isolated piRNAs were uridine in RNA/thymidine in the reverse-transcribed cDNA. (PDF) [file pgen.1006281.s008.pdf]

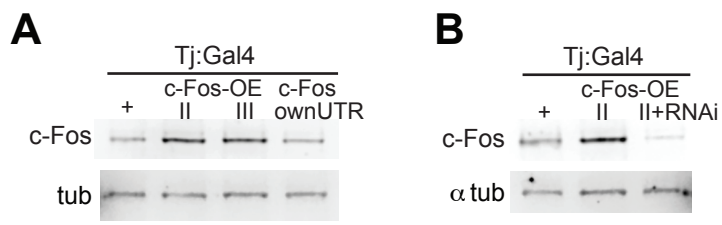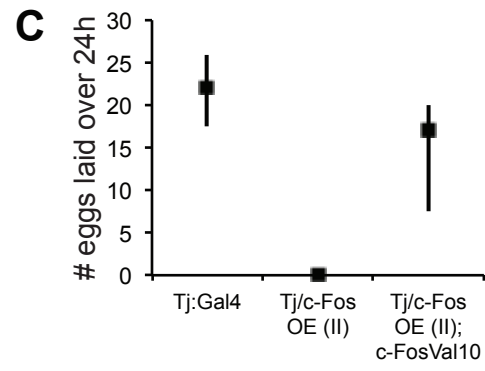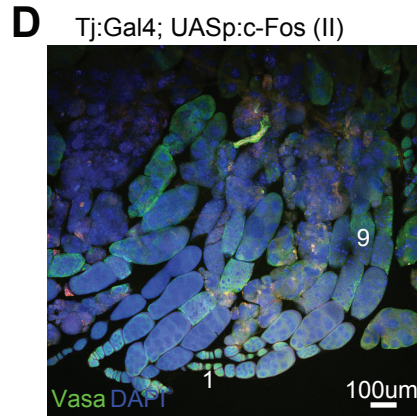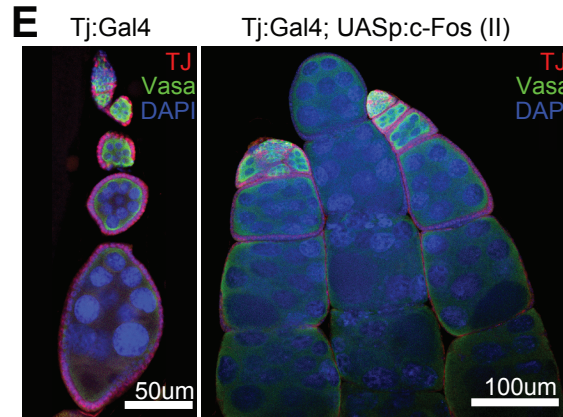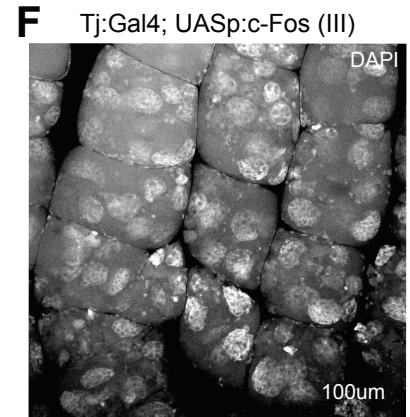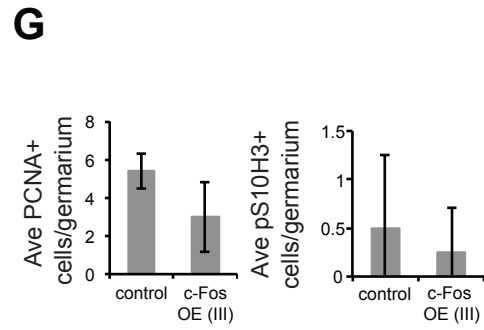

Supplement: S9 Fig — c-Fos and α-tubulin WB of ovarian extract from animals with Tj:Gal4 driving (A) (i) control, (ii) c-Fos overexpression II or III, (iii) c-Fos-ownUTR, (B) (iv) control, (v) c-Fos overexpression II, or (vi) c-Fos overexpression II; c-Fos shRNA-Val10. (C) Number of eggs laid by animals with Tj:Gal4 driving (iv) control, (v) c-Fos overexpression II, or (vi) c-Fos overexpression II; c-Fos shRNA-Val10 during a 24-h period. The black square represents the median value, and the lines indicate the twenty-fifth to seventy-fifth percentiles. (D) Vasa (green) IF in ovaries of Tj:Gal4 driving c-Fos overexpression II. Bar, 100 μm. The numbers indicate the first and ninth egg chamber in the highlighted ovariole. (E) Tj (red) and Vasa (green) IF of ovaries from Tj:Gal4 driving control or c-Fos overexpression II. Bars, 50 μm and 100 μm. White circles indicate oocyte in the same stage of the egg chamber. (F) DAPI-stained ovarioles in Tj:Gal4 driving c-Fos overexpression III. In later stages, germ cells appear necrotic. (G) Average PCNA-positive (indicating S phase) or phosphorylated serine 10 in histone H3-positive (indicating mitosis) ovarian cells per germarium from animals of Tj:Gal4 driving control or c-Fos overexpression (III). (PDF) [file pgen.1006281.s009.pdf]
