# Supplementary material for: c-Fos Repression by Piwi Regulates Drosophila Ovarian Germline Formation and Tissue Morphogenesis
Source: PLoS Genet. 2016 Sep 13;12(9):e1006281. doi: 10.1371/journal.pgen.1006281 (PMC5021354; doi:10.1371/journal.pgen.1006281)

A RNA-seq data to compare control and siRNAs in OSC lines:
Sienski et al., 2012, GSE41729
Ohtani et al., 2013, GSE 47006

FPKM (Fragments Per Kilobase of exon per Million exon-mapped fragments) values of c-Fos:

**OSCs**	**control**	**siPiwi**	**siArmi**	**siMael**
Sienski et al.	32.8258	43.3448	35.2957	31.5152
Ohtani et al.	54.1401	53.4492	56.0205	54.6634

**ovarian cells**	**wt -1**	**wt -2**	**wt -2**	***piwi* -1**	***piwi* -2**	***piwi* -3**	***c-Fos* OE-1**	***c-Fos* OE-2**	***c-Fos* OE-3**
our study	12.509	11.5167	13.667	34.1743	32.2791	36.0713	78.041	67.427	64.797

piwi/wt=3.4 FDR = 2.56x10^-5

B Unsupervised gene expression clustering

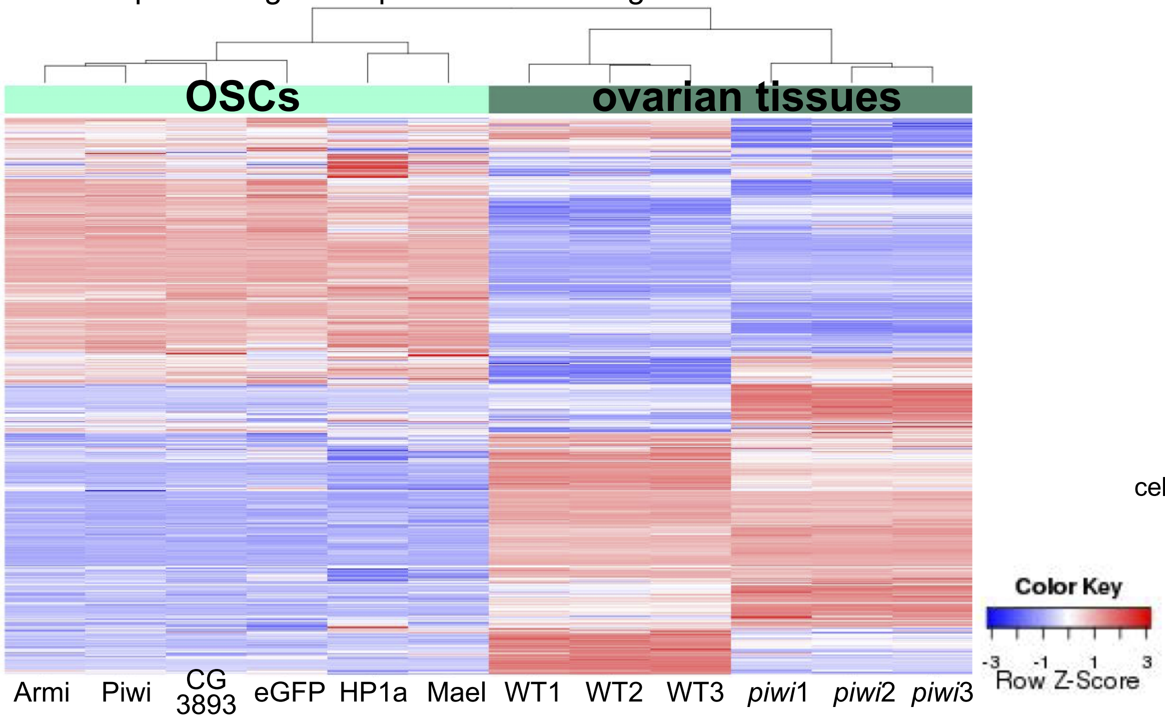

C Up-regulated in germline vs. OSCs

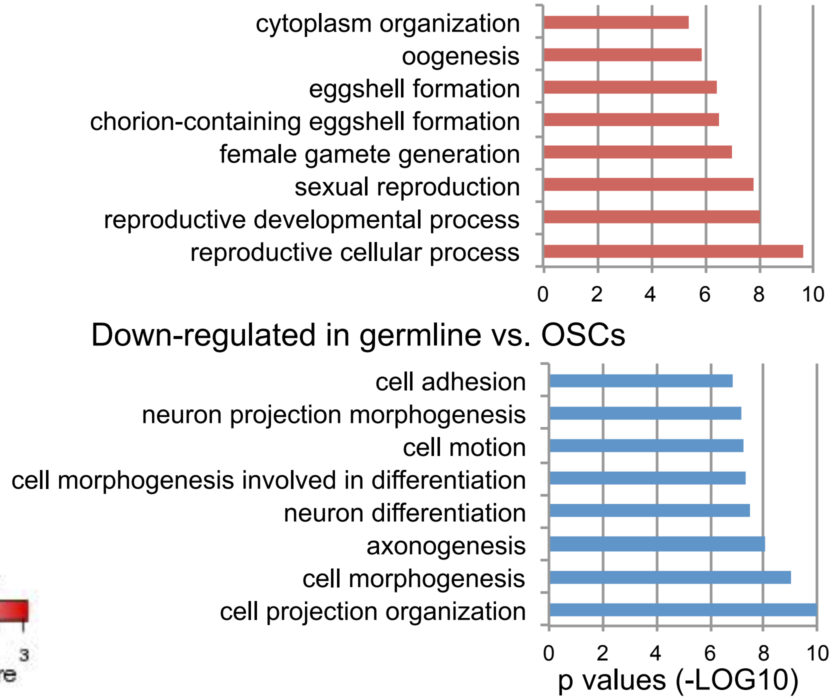

Supplement: S7 Fig — (A) RNA-seq data of OSCs from two studies were obtained. FPKM values of c-Fos from Sienski et al. were calculated by the investigator’s in-house perl script, and FPKM values of c-Fos from Ohtani et al. were calculated by using Cufflinks. Levels of c-Fos expression in OSCs were high and unchanged by knockdown of piRNA biogenesis factors. Our RNA-seq analysis, in triplicates, of wild type, piwi mutant, and c-Fos overexpressing ovarian cells. The level of c-Fos in piwi/wt ratio is ~3.4-fold at FDR (false discovery rate) of 2.45x10^-5^. (B) Unsupervised gene expression clustering of all genes in OSCs and germline tissues. In OSCs, siRNAs of genes were indicated. Blue-red heat map is of the z-scores. (C) Gene ontology analysis of the germline vs. the OSCs. P values in–LOG10. (PDF) [file pgen.1006281.s007.pdf]
