# Supplementary material for: c-Fos Repression by Piwi Regulates Drosophila Ovarian Germline Formation and Tissue Morphogenesis
Source: PLoS Genet. 2016 Sep 13;12(9):e1006281. doi: 10.1371/journal.pgen.1006281 (PMC5021354; doi:10.1371/journal.pgen.1006281)
Supplement: S1 Table — (PDF) [file pgen.1006281.s010.pdf]

Supplemental Table 1. Sequences of primers used for RT-qPCR

**Primers for RT-qPCR**			
Gypsy-F	gttcatacccttggtagtagc	Gypsy-R	caacttacgcatatgtgagt
Gypsy6-F	gacaagggcataaccgatactgtgga	Gypsy6-R	aatgattctgttccggacttccgtct
412-F	caccggtttggtcgaaag	412-R	ggacatgcctggtattttgg
rp49-F	ccgcttcaagggacagtatctg	rp49-F	atctcgccgcagtaaacgc
Rpl40-F	agggtggactccttctgaat	Rpl40-R	atcagcagcgtctgatcttc
c-Fos 1-F	cccagtgtgtcctcgatgt	c-Fos 1-R	acaaccacagcgacaacttt
c-Fos 1-R	aacaaccacagcgacaactt		
c-Fos ORFa-F	tgtacgaagcgttggagtcc	c-Fos ORFa-R	tgctacccaacgccattgat
c-Fos ORFb-F	tgttcgtggaggagctgttc	c-Fos ORFb-R	accggcaccttcaactttga
c-Fos UTRa-F	gggccgagtcaggattaagt	c-Fos UTRa-R	aagtgcttcgcggtttcctt
c-Fos UTRb-F	aaacctcttcattgcgcgtt	c-Fos UTRb-R	tcctgcaaatctacacgccc
GFP UTRa-F	aagcagcacgacttcttcaa	GFP UTRa-R	cggccatgatatagacgttg
GFP UTRb-F	cgtctatatcatggccgaca	GFP UTRb-R	ggactgggtgctcaggtagt
