# Supplementary material for: c-Fos Repression by Piwi Regulates Drosophila Ovarian Germline Formation and Tissue Morphogenesis
Source: PLoS Genet. 2016 Sep 13;12(9):e1006281. doi: 10.1371/journal.pgen.1006281 (PMC5021354; doi:10.1371/journal.pgen.1006281)
Supplement: S2 Table — (PDF) [file pgen.1006281.s011.pdf]

Supplemental Table 2. Sequences of primers used for ChIP-qPCR

Primer name	Primer sequence	Primer name	Primer sequence
c-Fos promoter-ChIP1f	ggttggagcgagacaaagat	c-Fos promoter-ChIP1r	gttgggaatgttctggcttt
c-Fos promoter-ChIP2f	ataatcgccaactggagagc	c-Fos promoter-ChIP2r	accatcttgaccctgtcctc
Intergenic-ChIP1f	ggcgagagggtcattaacat	Intergenic-ChIP1r	atttttcgggactccttgtg
Intergenic-ChIP2f	acagctggctaactgggagt	Intergenic-ChIP2r	ttggcataggtggaaatgaa
rp49 promoter-ChIP1f	ttccgaccacgttacaagaa	rp49 promoter-ChIP1r	aagaagcgcaaggagattgt
RPL40 promoter-ChIP1f	cgaaaaatcgcaataacgtg	RPL40 promoter-ChIP1r	ttcgacagaaacagctccac
